# Early In Ovo Sex Identification of Chicken Embryos Using a Dual-Stage Difference-Enhanced Spectral Fusion Network Based on Visible–Near-Infrared Transmission Spectroscopy

**DOI:** 10.3390/ani16172796

**Published:** 2026-09-05

**Authors:** Keqiang Li, Teng Chen, Dianzuo Yue, Chuanlong Guo, Sifeng Deng, Xianglong Li

**Affiliations:** 1 School of Mathematics and Information Technology, Hebei Normal University of Science and Technology, Qinhuangdao 066004, Chinayuedianzuo@163.com (D.Y.);; 2 Hebei Agricultural Data Intelligent Perception and Application Technology Innovation Center, Hebei Normal University of Science and Technology, Qinhuangdao 066004, China; 3 Hebei Key Laboratory of Specialty Animal Germplasm Resources Exploration and Innovation, Hebei Normal University of Science and Technology, Qinhuangdao 066004, China; 15203358192@163.com

**Keywords:** in ovo sex identification, chicken embryo, visible–near-infrared transmission spectroscopy, developmental spectral changes, spectral fusion, smart hatchery

## Abstract

Early in ovo sex identification is important for improving hatchery resource utilization; however, sex-related optical differences are weak during early embryonic development and exhibit clear stage dependence. In this study, longitudinal visible–near-infrared transmission spectra were acquired from 1600 fertilized pink-shelled eggs of Bashang Long-tailed chickens across four independent incubation batches during incubation days 1–7. Before model development, 200 eggs were reserved as a fixed internal hold-out test set, while the remaining 1400 eggs were used for exploratory incubation-day and feature screening, five-fold cross-validation, and model comparison. Among all 21 dual-day combinations, D2 + D5 achieved the highest exploratory validation AUC of 0.951. The selected scheme required separate measurements on D2 and D5, with the final sex classification completed on D5. Further incorporation of the signed difference spectrum ΔS and relative change rate R increased the AUC to 0.965, demonstrating the incremental discriminative value of longitudinal developmental-change information. After the input scheme had been fixed, DSSF-Net achieved the numerically highest performance among the evaluated models under unified egg-level stratified five-fold cross-validation, with an accuracy of 0.915 ± 0.015, an AUC of 0.974 ± 0.012, and an F1-score of 0.915 ± 0.014. The final model achieved an accuracy of 0.915 and an empirical AUC of 0.966 (95% CI: 0.940–0.987) on the fixed internal hold-out test set. Complete four-round leave-one-batch-out validation yielded mean accuracy and AUC values of 0.846 ± 0.014 and 0.926 ± 0.012, respectively, indicating that the longitudinal spectral information from D2 + D5 maintained relatively stable discriminative performance across incubation batches within the investigated population. These results demonstrate the feasibility of longitudinal spectral modeling for early sex identification in fertilized pink-shelled eggs of Bashang Long-tailed chickens. Systematic offline validation was completed under laboratory conditions, including evaluation on the fixed internal hold-out test set and four-round leave-one-batch-out validation, supporting the effectiveness and batch-level stability of D2 + D5 longitudinal developmental spectra. Post hoc wavelength-level interpretation further identified 594–620 nm and 660–675 nm as two candidate spectral regions with relatively high predictive value. Collectively, these findings provide a reliable longitudinal spectral modeling framework for early non-destructive in ovo sex identification and establish an experimental and methodological foundation for wavelength selection, device development, and subsequent engineering validation of multispectral detection systems under hatchery conditions.

## Simple Summary

Identifying the sex of chicken embryos before hatching can help hatcheries reduce unnecessary incubation and improve animal-welfare management. However, optical differences between male and female embryos are weak during the first days of incubation. This study measured visible and near-infrared light transmitted through the same eggs from the first to the seventh day and investigated whether changes between developmental stages could improve sex identification. Measurements from the second and fifth days provided the most useful complementary information. A model combining these two measurements with their developmental changes correctly classified 91.5% of eggs in an internal hold-out test set. When evaluated on previously unseen incubation batches, it achieved an average accuracy of 84.6%, showing that differences among batches remain an important practical challenge. These findings demonstrate that repeated measurements of the same egg can support non-destructive sex identification by the fifth day of incubation and identify specific wavelength ranges for developing simpler detection equipment. Further validation across breeds, shell colors, hatcheries, and devices is required before commercial application.

## 1. Introduction

The layer industry is a vital component of modern poultry production systems. Production efficiency, resource utilization, and animal welfare directly affect the sustainable development of the sector [1,2]. In egg production, male and female chicks differ substantially in economic value: female chicks proceed to rearing and laying stages, whereas male chicks in layer lines generally have limited commercial value. Producers therefore rely on post-hatch manual sexing to separate male and female chicks. Because sex cannot be determined until hatch, all eggs must undergo the full incubation process, resulting in continued expenditure of energy, labor, and management resources. Moreover, manual post-hatch sexing is difficult to integrate into large-scale, automated, and precision hatchery systems. If the sex of chicken embryos could be identified rapidly, non-destructively, and accurately during early incubation, eggs could be sorted before substantial embryonic development, thereby optimizing hatchery resource allocation, reducing ineffective incubation, and providing key technical support for intelligent incubation and precision poultry production [3].

Several technical routes have been developed for in ovo sex identification of chicken embryos, including biomarker detection, optical non-destructive detection, machine vision, and multi-source information fusion [4]. Among these, optical non-destructive methods can acquire internal egg information without breaching the eggshell or removing biological material, making them potentially suitable for low-intervention and automated detection in commercial hatcheries [5]. Biomarker-based approaches using hormones, DNA, metabolites, or embryonic fluids provide strong biological evidence for sex discrimination and can achieve high accuracy in some studies; however, they often require egg opening, sampling, or complex chemical analysis, resulting in complicated workflows, limited detection speed, increased contamination risk, and difficulty in online deployment [6].

Visible–near-infrared (Vis-NIR) spectroscopy, hyperspectral imaging, Raman spectroscopy, and fluorescence spectroscopy have been widely applied to fertilization detection, live-embryo identification, embryonic development monitoring, and in ovo sex determination [7,8,9,10,11]. The underlying principle is that embryonic development continuously alters internal tissue structure, vascular networks, heme-related absorption, metabolic activity, and light-scattering pathways, which produce detectable differences in transmission or reflection spectra. Hyperspectral imaging, in particular, provides both continuous spectral and spatial information, showing considerable potential for non-destructive sex identification [8]. Nevertheless, high-accuracy results are often obtained during mid-to-late incubation, when embryonic volume, vascular structures, and internal optical contrasts are more pronounced. During early incubation, embryonic structures are not fully developed, and spectral differences between male and female embryos are weak and unstable, which remains a key bottleneck [4,9,10,11,12].

Machine vision methods mainly exploit vascular morphology, internal shadow changes, blastoderm development, or shell appearance [13,14]. These approaches are relatively inexpensive and can be integrated with automated imaging systems, but their performance is easily affected by shell color, shell thickness, translucency, imaging angle, and illumination conditions. For early embryos, vascular and tissue structures are not yet prominent, making stable sex discrimination based on image features alone difficult. Consequently, increasing attention has been paid to combining spectral, image, and morphological features to enhance the representation of weak sex-related signals [15]. However, multi-source fusion is not equivalent to simply increasing input variables. Without analyzing embryonic developmental stages and information complementarity, direct fusion of heterogeneous features may introduce redundancy and even reduce model stability and interpretability [16].

For spectral modeling, partial least squares discriminant analysis (PLS-DA), support vector machines (SVMs), random forests (RFs), and extreme gradient boosting (XGBoost) have been widely used for high-dimensional spectral classification [17,18,19,20]. These methods perform reasonably well under small-to-medium sample sizes but usually depend on manual feature selection and fixed input structures, limiting their ability to capture complex nonlinear band relationships, cross-stage variations, and individual differences [21]. In recent years, one-dimensional convolutional neural networks (1D-CNNs), recurrent neural networks, and Transformer models have been introduced to automatically extract local band patterns, sequential dependencies, and long-range spectral associations [22,23,24,25,26,27]. Although these methods improve spectral feature learning, model performance in early embryo sex identification is still constrained by weak sex-related signals, uneven stage-wise information, and redundancy across multiple incubation days.

More importantly, chicken embryo sex identification is not a conventional spectral classification problem at a single time point, but a dynamic discrimination task embedded in continuous embryonic development. During early incubation, embryonic tissue, vascular networks, metabolic status, and internal scattering structures change rapidly, and spectra from different incubation days do not carry equal discriminative value. One day may reflect early sex-related optical responses, whereas another may contain more pronounced tissue development or vascular changes [12,13]. Using spectra from only one incubation day may lead to insufficient stage information, whereas directly concatenating spectra from multiple days may ignore developmental logic and make it difficult for models to distinguish the contributions of static spectral states and dynamic developmental changes to sex discrimination. Therefore, the core challenge in early embryo sex identification is not merely selecting a detection device or classifier, but identifying complementary developmental stages and transforming cross-stage spectral changes of the same egg into biologically meaningful and learnable feature representations.

In commercial hatcheries, the practical value of early in ovo sex identification depends not only on classification performance but also on the timing of detection, the number of measurements, egg-handling procedures, and the potential consequences of misclassification. Compared with post-hatch sex determination, obtaining sex information during early incubation may help reduce unnecessary continued incubation and post-hatch handling. Single-day detection offers the advantages of a simpler workflow, fewer handling operations, and potentially lower implementation costs; however, it may not adequately capture the weak and stage-dependent sex-related information associated with embryonic development. Multi-time-point detection can exploit longitudinal developmental changes within the same egg and thereby improve the reliability of sex discrimination, but it also increases the requirements for egg handling, identity tracking, longitudinal data linkage, and equipment configuration. Therefore, the choice of detection strategy should not be based solely on minimizing the number of measurements or maximizing AUC, but should balance predictive performance, animal-welfare benefits, misclassification risks, and the practical costs of hatchery implementation.

Previous optical studies have provided an important foundation for early non-destructive in ovo sex identification [4]. Zhu et al. [12] collected UV/Vis transmission spectra from 210 eggs over incubation days 0–15 and developed an extreme learning machine model, with the best discrimination performance obtained on D7. Pan et al. [13] acquired hyperspectral transmission images of 94 eggs on D2, D4, D6, D8, D10, and D12 within 400–1000 nm and compared SVM, PLS-DA, and ANN models, reporting the best prediction accuracy of 82.86% for the ANN model on D10. These studies demonstrated that sex-related spectral information varies with embryonic development and that the selection of incubation day substantially affects classification performance. However, the measurements from different days were mainly evaluated as separate day-specific inputs. The complementary value of paired early developmental stages was not systematically examined, and all 21 possible dual-day combinations within D1–D7 were not comprehensively compared. Moreover, the longitudinal information from the same egg was not explicitly decomposed into stage-specific spectral states, signed change magnitudes, and relative change trends, making it difficult to determine whether performance gains arise from one dominant stage or from complementary developmental changes between stages. Although established methods such as CARS and SPA can support spectral-variable selection, and SHAP and local explanation approaches can provide post-hoc model interpretation [16,28,29,30,31,32,33], their coordinated use to connect key incubation days, longitudinal change features, and predictive wavelength regions remains limited in early in ovo sex identification.

To address these issues, this study used fertilized pink-shell eggs of Bashang Long-tailed chickens to construct a continuous Vis-NIR transmission spectral dataset during early incubation and proposed a dual-stage difference-enhanced spectral fusion network (DSSF-Net) for early non-destructive sex identification. Spectral data were collected continuously from the same eggs on incubation days 1–7 (D1–D7) to analyze sex-related transmission responses and their distributions across stages. Single-day, dual-day, and multi-day spectral combinations were then constructed to identify key time windows with favorable recognition performance and sampling cost. Signed difference spectrum and relative change rates were further derived to characterize longitudinal changes of the same egg across developmental stages. Finally, stage-specific spectral feature extraction, developmental difference enhancement, and stage-aware attention fusion were used to adaptively integrate static stage information and dynamic developmental changes.

The main contributions of this study are fourfold. First, based on continuous D1–D7 data, the contributions of different incubation days and their combinations to embryo sex identification were systematically evaluated, providing experimental evidence for selecting early non-destructive detection windows. Second, difference spectra and relative change rates were introduced to extend sex identification from single-stage static discrimination to cross-stage developmental-change discrimination, enhancing the model’s ability to capture weak sex-related signals. Third, DSSF-Net was proposed to adaptively fuse stage-specific spectral features and their change features through stage-specific branches, a developmental difference enhancement branch, and a stage-aware attention mechanism, thereby reducing redundancy caused by simple concatenation. Fourth, model comparison, cross-batch validation, ablation experiments, and wavelength-level interpretation were conducted to verify the effectiveness, stability, and interpretability of the proposed method and to provide candidate wavelength regions for subsequent reduced-band modeling and independent hardware validation.

## 2. Materials and Methods

### 2.1. Egg Samples and Incubation Management

Fertilized pink-shell eggs of Bashang Long-tailed chickens were used in this study. Chicks of this breed exhibit obvious sex-linked plumage color differences at hatch, allowing sex determination based on down color at day of hatch. Therefore, post-hatch plumage-based sexing results were used as embryonic sex labels to ensure reliable model training and evaluation.

Before incubation, the eggs were surface-disinfected using a 0.005% benzalkonium bromide working solution prepared by diluting the 5% stock solution at a ratio of 1:1000. After disinfection, the eggs were naturally air-dried before identification and incubation. After natural air-drying, each egg was uniquely numbered and assigned a data record covering the entire incubation period. Eggs were then incubated in an automatic egg incubator (HC-1056A, Liucan, Dezhou, China) at (37.8 ± 0.1) °C and 60–65% relative humidity, with automatic turning every 2 h. On day 18, eggs were transferred, turning was stopped, and relative humidity was increased to 65–70% until hatch. Stable ventilation was maintained throughout incubation, and equipment operation was checked regularly to reduce environmental fluctuations affecting embryonic development and spectral responses.

To capture longitudinal spectral changes during early embryonic development, spectra were acquired every 12 h from D1 to D7, resulting in 14 time-point spectra per egg. The two spectra collected within each incubation day were averaged wavelength by wavelength to generate one daily representative spectrum, yielding seven daily spectra (D1–D7) for subsequent analyses. Each complete acquisition session for 600 eggs lasted approximately 2 h and 20 min and was performed by two trained operators using one Vis–NIR measurement system. The average operating time was approximately 12.5 s per egg, including identification verification, removal, positioning, acquisition of three consecutive spectra, data storage, and return to the incubator. Eggs were removed individually, remained outside the incubator for no more than 1 min, and were measured on a platform adjacent to the incubator. Temperature and relative humidity were continuously monitored, and the incubator door was kept closed between egg transfers. Egg orientation, measurement distance, illumination conditions, instrument settings, and acquisition order were kept consistent. Because the interval between the first and last eggs was approximately 2.3 h, D1–D7 represent standardized acquisition windows rather than identical instantaneous developmental ages; however, using the same egg sequence at each session maintained an approximately 12-h interval between successive measurements of each individual egg.

To reduce dependence on a single incubation run and evaluate inter-batch stability, four independent incubation batches were conducted using unified egg-screening, incubation, and spectral-acquisition procedures. Before incubation, eggs with cracked shells, severe surface contamination, abnormal morphology, or other visible defects were excluded, and 600 intact, clean, and normally shaped eggs were placed in incubation in each batch. Each egg was assigned a unique identification number, and longitudinal spectral data were collected before embryo sex was known. Among the eggs placed in incubation, 60, 54, 66, and 48 eggs were identified as infertile; 30, 27, 33, and 25 experienced embryonic mortality; and 10, 12, 11, and 9 remained unhatched in Batches 1–4, respectively. After hatching, chick sex was independently determined by two trained observers according to the sex-linked plumage characteristics of day-old chicks. Both observers were blinded to the spectral data and model predictions. Samples for which the two observers disagreed were further assessed by a third experienced observer, and those for which a consensus label could not be obtained were classified as having uncertain sex labels and excluded. Each confirmed sex label was linked to the corresponding egg and its longitudinal spectral records using the unique egg identification number. Among the successfully hatched eggs, 3, 2, 3, and 2 samples were excluded because of incomplete spectral records, while 2, 1, 2, and 1 samples were excluded because of uncertain sex labels. Before class balancing, the eligible male/female sample numbers were 241/254, 261/243, 235/250, and 266/249 for Batches 1–4, respectively. Finally, 200 male and 200 female eggs were randomly selected from the eligible samples of each batch at the egg-ID level before dataset partitioning and model development, resulting in a balanced dataset of 1600 eggs across the four batches.

Among the eggs not classified as infertile, 500 of 540, 507 of 546, 490 of 534, and 518 of 552 eggs reached hatch in Batches 1–4, respectively. The corresponding descriptive hatchability rates among fertile eggs were 92.6%, 92.9%, 91.8%, and 93.8%, with an overall rate of 92.8% (2015/2172). No contemporaneous farm hatchability data or incubator-control data were available, and no non-measured or minimally handled control group was included. Therefore, these descriptive hatchability results cannot establish that repeated handling, temporary removal from incubation, or halogen illumination had no effect on embryonic mortality or hatchability.

Measurement order and incubator positions were not randomized. Because each complete acquisition session lasted approximately 2.3 h, eggs measured at the beginning and end of the same session were not at exactly the same developmental time. Using the same fixed measurement order maintained an approximately 12-h interval between successive measurements of each individual egg, but it could not completely eliminate residual within-session developmental-time variation. In addition, spatial differences within the incubator could have indirectly affected embryonic development, and the fixed acquisition order may have allowed residual time-dependent instrumental variation. These effects were reduced through standardized acquisition, repeated calibration, and consistent egg-level longitudinal tracking, but they could not be completely excluded.

### 2.2. Spectral Acquisition and Preprocessing

A Vis–NIR transmission spectroscopy system was used to acquire egg spectral data. The system consisted of an ICL-B1620 CCD camera (Imperx, Boca Raton, FL, USA), a T3 miniature compound spectral sensor (Daohuozhe, Hangzhou, China), an adjustable-focus lens (Z118M0736C6M, Yulufa, Quanzhou, China), a 150 W halogen light source (15 V/150 W, Philips, Shenzhen, China), and a data-acquisition computer (Think Station P620, Lenovo, Beijing, China). The egg holder and sample platform were custom-built noncommercial components designed and fabricated in our laboratory at Hebei Normal University of Science and Technology, Qinhuangdao, China, and therefore had no commercial manufacturer or model designation. The effective spectral range was 340–1020 nm, with a 1 nm sampling interval (681 wavelength points) and a spectral resolution of approximately 2.8 nm. The halogen lamp was preheated for 30 min before formal acquisition to ensure stable output [34,35,36,37].

**Figure 1:**
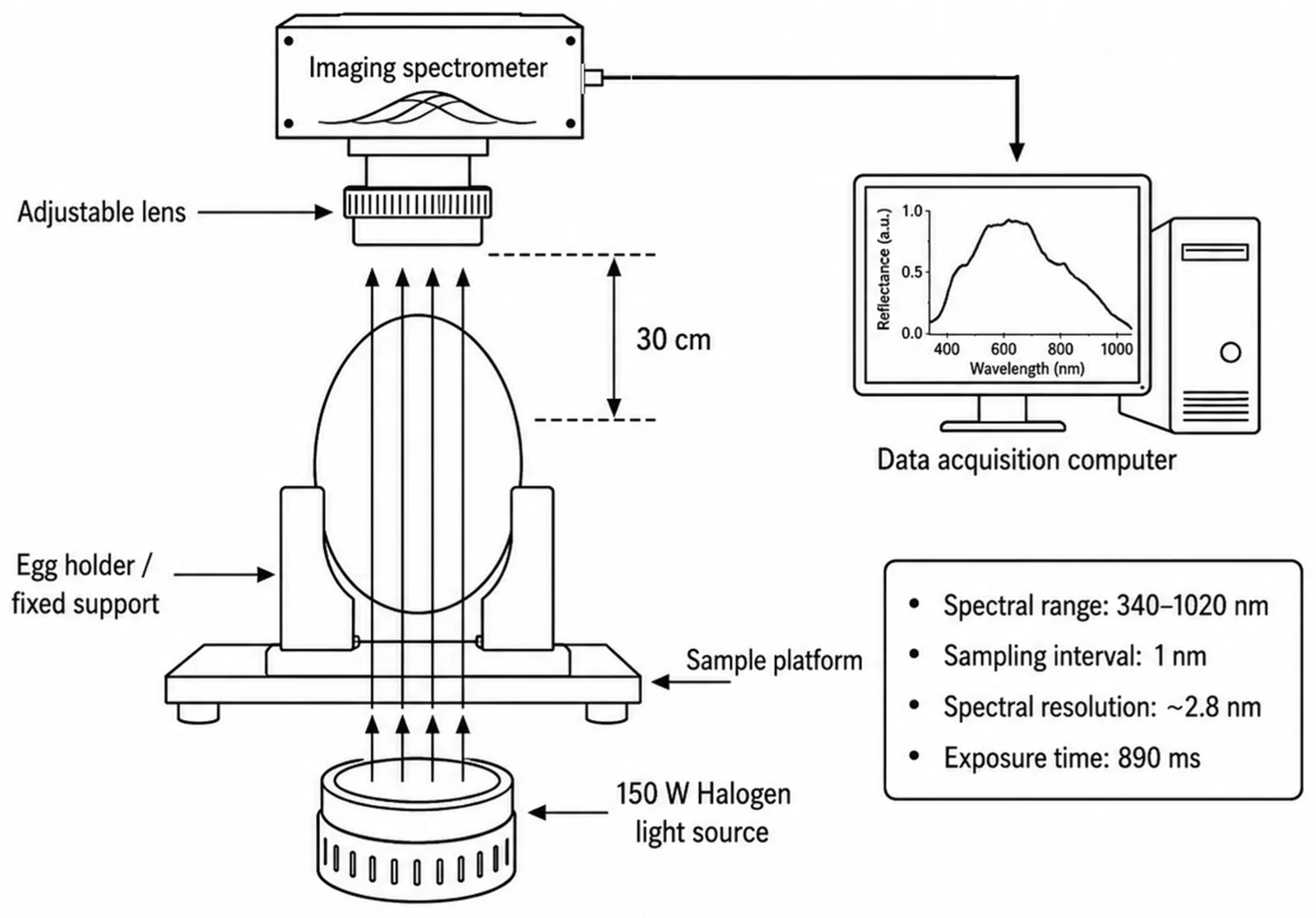
Schematic diagram of egg spectral acquisition.

Spectra were acquired in transmission mode. As shown in Figure 1, each egg was fixed with the blunt end up and the pointed end down. The halogen lamp was placed below the egg and the camera above it. The lens-to-egg distance was maintained at 30 cm, and the exposure time was set to 890 ms. At each sampling event, three consecutive spectra were acquired using an exposure time of 890 ms per measurement, corresponding to a total nominal detector integration time of 2.67 s per egg. Including egg handling, positioning, identification verification, data storage, and return to the incubator, the average operating time was approximately 12.5 s per egg. White and dark reference corrections were performed before each continuous acquisition sequence, and recalibration was conducted every 30 min to reduce effects of dark current, source fluctuation, and system noise. Embryonic development is closely related to egg optical responses, and previous studies support continuous day-wise sampling schemes [38,39,40].

Raw spectral responses were converted into relative transmittance using white- and dark-reference correction:

(1) $$T\left(\lambda \right)=\frac{I_{s}\left(\lambda \right)-I_{d}\left(\lambda \right)}{I_{w}\left(\lambda \right)-I_{d}\left(\lambda \right)}$$

$T\left(\lambda \right)$ was retained as a dimensionless relative-transmittance ratio rather than being converted to percentage transmittance or absorbance. After dark-reference correction, values of 0 and 1 corresponded nominally to the dark and white reference levels, respectively. $I_{s}\left(\lambda \right)$, $I_{d}\left(\lambda \right)$, and $I_{w}\left(\lambda \right)\;$ denote the sample, dark-reference, and white-reference intensities at wavelength λ, respectively. After correction, the valid egg-transmission region was separated from the background, and the pixel responses within this region were averaged wavelength by wavelength to obtain one mean transmission spectrum for each egg. Spatial texture, shape, and morphological information from the hyperspectral images were not used as model inputs.

At each 12-h acquisition time point, three consecutive spectral measurements were obtained for each egg and first averaged to generate one representative time-point spectrum. The two representative time-point spectra acquired within the same incubation day were then averaged wavelength by wavelength to generate the daily representative spectrum used for modeling.

Only eggs with complete spectral records at all 14 acquisition time points were eligible for inclusion in the final dataset. Eggs with any missing time-point measurement were excluded before sex-stratified random sampling, and no interpolation, imputation, or substitution using a single within-day measurement was performed. Consequently, no missing spectral values remained in the 1600 eggs included in the final analysis.

No additional smoothing, derivative transformation, standard normal variate (SNV) transformation, or multiplicative scatter correction (MSC) was applied in the final modeling pipeline. The wavelength range of 340–1020 nm was predefined as the effective spectral range after instrument calibration. All 681 wavelength points within this range were retained, and no additional noisy-band removal was performed. No statistical outlier removal based on spectral distance, class labels, or prediction errors was conducted.

For scale-sensitive classification models, wavelength-wise Z-score standardization was performed as follows:

(2) $$x_{i,\lambda }^{\prime }=\frac{x_{i,\lambda }-\mu_{\lambda ,\mathrm{train}}}{\sigma_{\lambda ,\mathrm{train}}}$$

where $\mu_{\lambda ,\mathrm{train}}\;$ and $\sigma_{\lambda ,\mathrm{train}}\;$ denote the mean and standard deviation of wavelength λ, estimated exclusively from the corresponding training set or training fold. The validation set, outer evaluation fold, independent test set, and external batch were transformed using these training-derived parameters.

### 2.3. Construction of Single-Day, Multi-Day, and Developmental-Change Spectral Datasets

To analyze the contribution of spectral information from different incubation stages and to identify day combinations suitable for early non-destructive detection, single-day, dual-day, multi-day, and developmental-change spectral datasets were constructed from continuous D1–D7 spectra of the same eggs [12,13].

First, spectra from incubation days 1–7 were extracted separately to construct seven single-day datasets (D1–D7). Each dataset contained 1600 samples with 681 wavelength points per sample. The single-day spectral feature matrix for day d is defined in Equation (3).

(3) $$X^{\left(d\right)}={\left[{\bar{S}}_{1,d},{\bar{S}}_{2,d},\ldots ,{\bar{S}}_{N,d}\right]}^{T}\in R^{N\times 681}$$

where $X^{\left(d\right)}$ denotes the spectral feature matrix on day d and N is the number of valid eggs. Models trained under identical data partitioning, preprocessing, and hyperparameter settings were used to evaluate the independent discriminative ability of each incubation stage.

Based on single-day analysis, dual-day spectral combination datasets were further constructed. For any two incubation days $d_{1}$ and $d_{2}$ ($d_{1}$ < $d_{2}$), the spectral vectors of the same egg were concatenated.

(4) $$X_{i}^{\left(d_{1},d_{2}\right)}=\left[{\bar{S}}_{i,d_{1}},{\bar{S}}_{i,d_{2}}\right]$$

where $X_{i}^{\left(d_{1},d_{2}\right)}$ represents the fused spectral feature of the i-th egg. Each dual-day sample contained 2L features.

To evaluate whether classification performance continued to improve as the number of sampling days increased, multi-day datasets were constructed. For a combination containing k incubation days with index set S = {$t_{1}$, $t_{2}$, …, $t_{k}$}, the corresponding fused feature is defined in Equation (5).

(5) $$X_{i}^{\left(D\right)}=\left[{\bar{S}}_{i,d_{1}},{\bar{S}}_{i,d_{2}},\ldots ,{\bar{S}}_{i,d_{k}}\right]$$

where $D=\left\{d_{1},d_{2},\ldots ,d_{k}\right\}$ denotes the selected set of incubation days, and k denotes the number of incubation days included in the combination. The feature dimension of each sample is 681×k. Experiments focused on consecutive-day and interval-day combinations, including D1 + D2 + D3, D2 + D3 + D4, D2 + D5 + D7, D1–D5, and D1–D7. By comparing classification performance, input dimensionality, and data collection cost across different combinations, we analyzed whether model performance continued to improve as the number of sampling days increased, and screened combination schemes that balance recognition performance.

The single-day, dual-day, multi-day, and developmental-change feature datasets constructed in this section were primarily used for exploratory screening. All candidate incubation-day and feature combinations were evaluated using the same egg-level data partition and SVM classifier, with only the input day or feature combination being varied. The validation AUC obtained at this stage was used to compare the relative performance of the candidate schemes and to determine the key incubation days and input features for subsequent model development; it was not regarded as an estimate of the final model’s generalization performance. The specific partitioning procedure and sample sizes for each data subset are described in Section 2.5.2.

### 2.4. Proposed DSSF-Net Model

To address weak early sex-related spectral signals, insufficient representation from a single sampling stage, and redundancy introduced by direct multi-stage concatenation, a dual-stage difference-enhanced spectral fusion network (DSSF-Net) was proposed. The model does not fix incubation days a priori; instead, it accepts preprocessed spectra from one or more sampling stages. For single-stage input, the model performs stage-specific spectral feature extraction and classification. For two or more stages, the developmental difference enhancement branch and stage-aware attention fusion module are activated to jointly exploit static spectral states and dynamic developmental changes.

**Figure 2:**
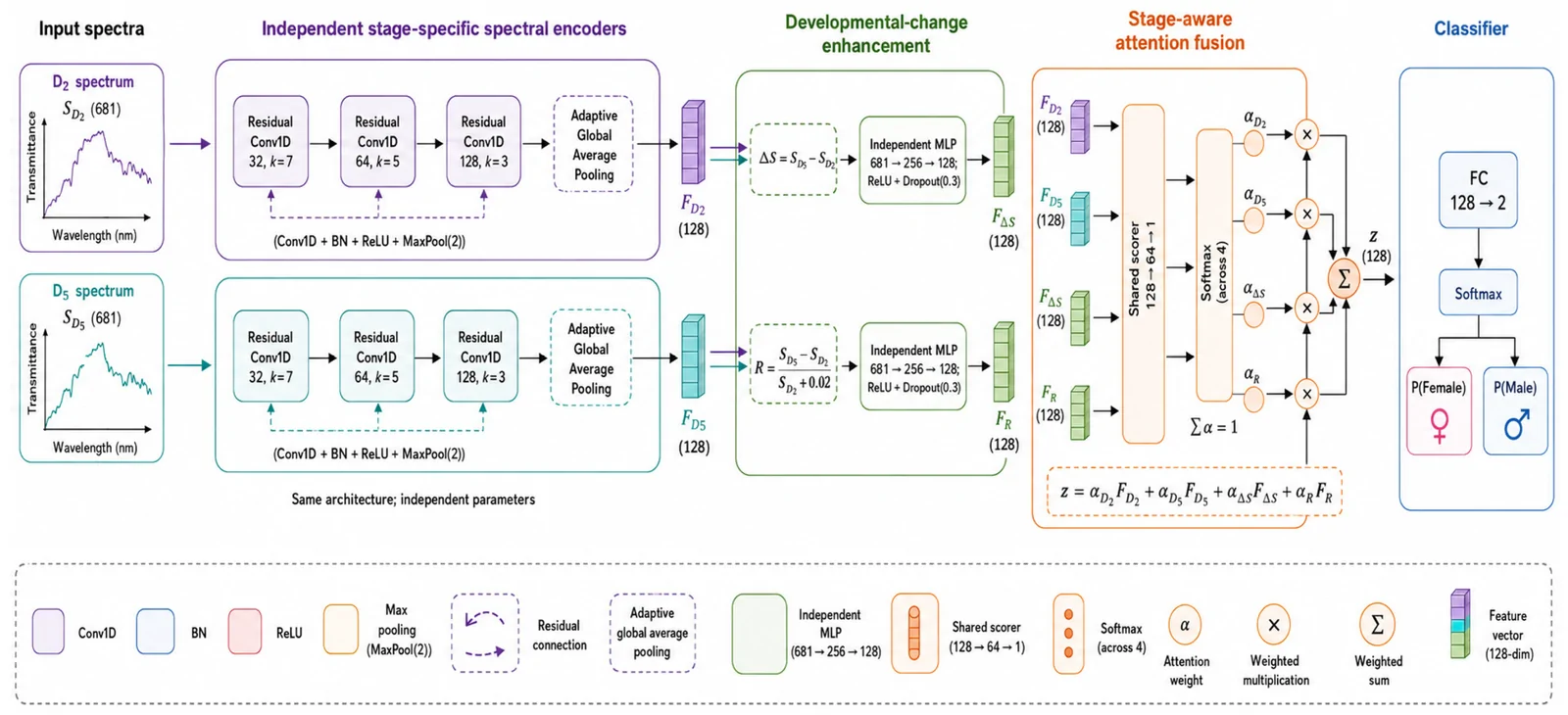
Overall architecture of DSSF-Net.

The core idea of DSSF-Net is to transform multi-stage spectral fusion from simple concatenation at the raw-variable level to adaptive fusion at the feature level. As shown in Figure 2, the model first encodes spectra from different stages, then constructs developmental difference features based on cross-stage changes of the same egg, and finally adaptively quantifies the contributions of stage features and change features to sex discrimination. This structure is compatible with single-day, dual-day, and multi-day inputs without predefining the optimal incubation day.

#### 2.4.1. Generalized Spectral Input and Model Workflow

The complete DSSF-Net workflow consists of three steps: (1) stage-specific spectral feature extraction; (2) when m ≥ 2, computation of signed difference spectrum and relative change rates between ordered stages through the developmental difference enhancement module; and (3) stage-aware attention fusion of stage and change features followed by classification.

For egg i, let the preprocessed spectra collected at stage set {$t_{1},t_{2}\ldots t_{m}$} be denoted as $X_{i}^{T}$, where m is the number of stages and each spectrum contains L wavelength points determined by the effective spectral range and sampling interval. The corresponding model input is defined in Equation (6).

(6) $$x_{i}^{T}={\left[x_{i,t_{1}},x_{i,t_{2}},\ldots ,x_{i,t_{m}}\right]}^{T},\;\;\;\;\;\;\;\;\;\;\;\;\;\;\;x_{i,t_{j}}\in R^{L}$$

where m = 1 indicates single-stage input and m ≥ 2 indicates multi-stage input. To prevent data leakage, all spectral records associated with the same egg number were consistently assigned to the training, validation, or test set.

#### 2.4.2. Stage-Specific Spectral Feature Extraction Module

**Figure 3:**
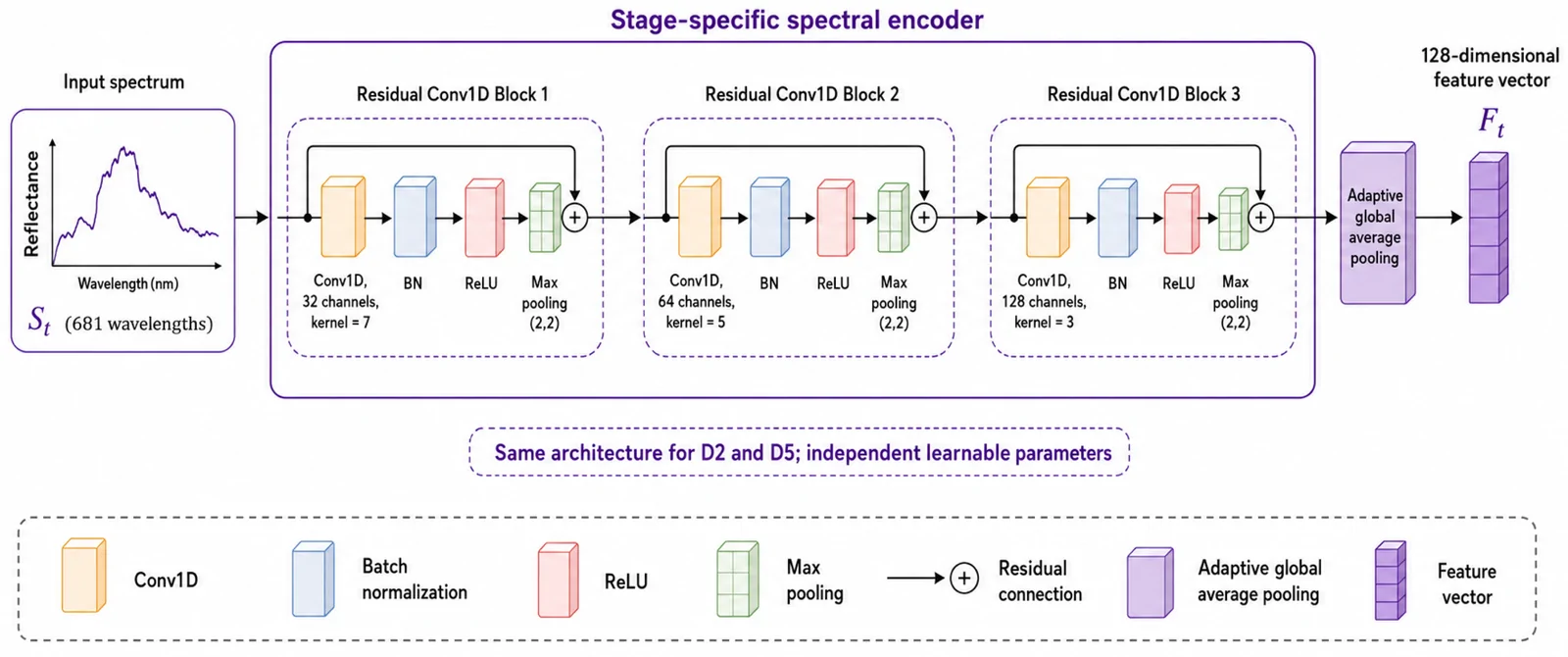
Architecture of the stage-specific spectral feature extraction module.

As shown in Figure 3, the final DSSF-Net contained two independent stage-specific spectral encoders for the D2 and D5 spectra. Each encoder received a 681-dimensional spectral vector and consisted of three residual Conv1D blocks with output channel numbers of 32, 64, and 128 and kernel sizes of 7, 5, and 3, respectively. In the main branch of each block, Conv1D was followed by batch normalization, ReLU activation, and max pooling with a kernel size and stride of 2. Because the channel number and spectral-sequence length changed between successive blocks, a 1 × 1 convolution and corresponding downsampling were applied to the projection shortcut before element-wise addition [26]. After the third residual block, adaptive global average pooling converted the feature map into a 128-dimensional stage-specific feature vector. The D2 and D5 encoders used identical architectures but maintained independent learnable parameters, allowing them to characterize stage-specific spectral patterns separately. The two stage-specific encoders contained 92,416 trainable parameters in total.

(7) $$F_{t}=E_{t}\left(S_{t};\theta_{t}\right),\;\;\;\;\;\;\;F_{t}\in R^{128}$$

where $S_{t}$ denotes the 681-dimensional spectrum acquired at stage t, $E_{t}$ represents the corresponding stage-specific encoder, and $\theta_{t}$ denotes its learnable parameters. Although $E_{D2}$ and $E_{D5}$ shared the same architecture, their parameters were learned independently.

#### 2.4.3. Developmental Difference Enhancement Module

**Figure 4:**
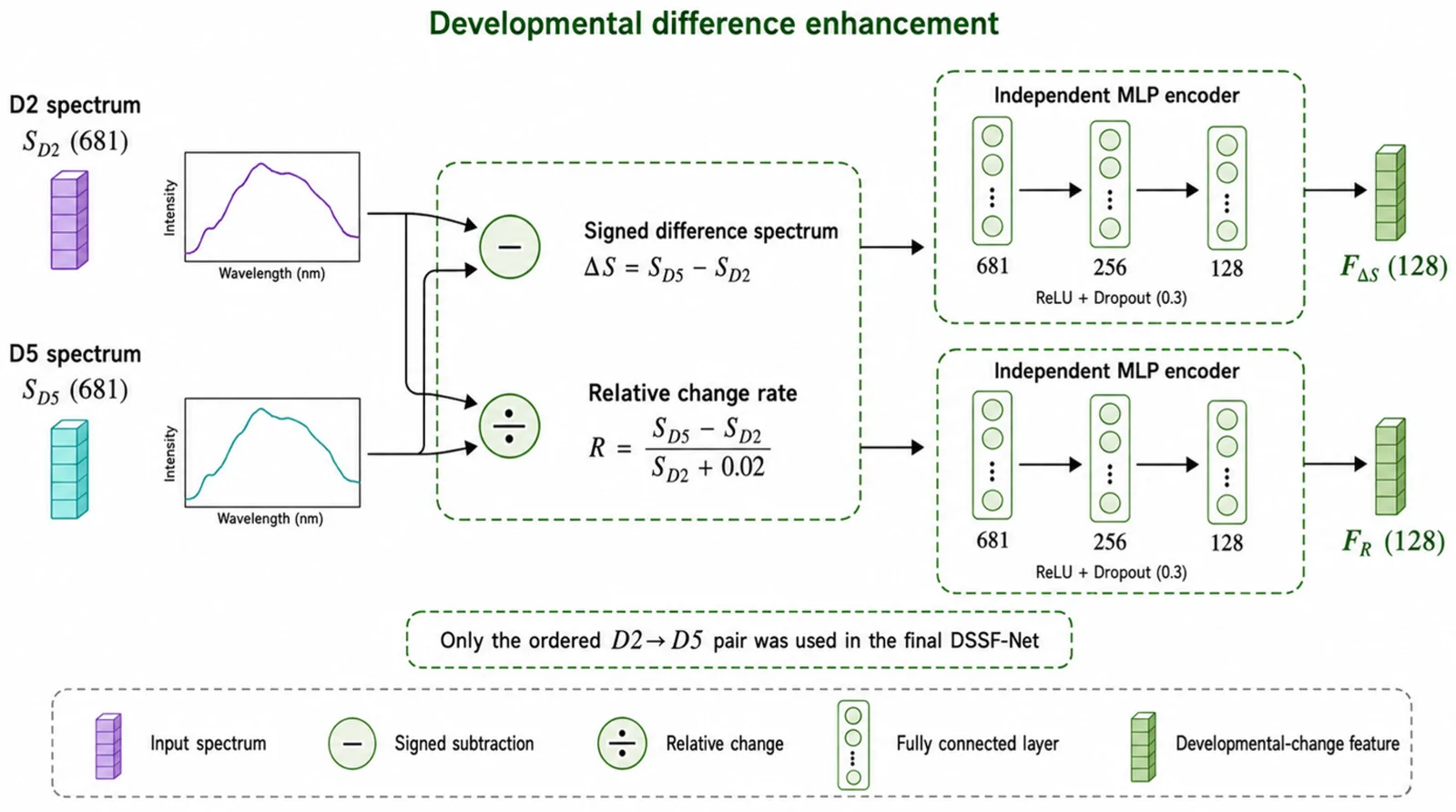
Architecture of the developmental difference enhancement module.

Early sex-related spectral differences are usually weak, and static spectra from a single stage are easily affected by shell thickness, initial transmission intensity, individual variation, and measurement noise. To emphasize self-changes during incubation, DSSF-Net includes a developmental difference enhancement module, as shown in Figure 4. In the final DSSF-Net, only the ordered D2→D5 pair was used to construct the developmental-change inputs. For each egg, the signed difference spectrum ΔS and relative change rate R were calculated wavelength by wavelength from the white- and dark-reference-corrected D2 and D5 relative-transmittance spectra before Z-score standardization.

(8) $$\Delta S_{i}=S_{i,D_{5}}-S_{i,D_{2}}$$

(9) $$R_{i}=\frac{S_{i,D_{5}}-S_{i,D_{2}}}{S_{i,D_{2}}+0.02}$$

where $S_{i,D_{2}}$ and $S_{i,D_{5}}$ denote the white- and dark-reference-corrected relative-transmittance spectra of the i-th egg acquired on D2 and D5, respectively. The constant 0.02 was defined on this dimensionless relative-transmittance scale and was used to reduce numerical instability when the D2 transmittance approached zero. After ΔS and R were calculated, D2, D5, ΔS, and R were standardized separately using training-data statistics before being supplied to the classification models.

The signed difference spectrum ΔS and relative change rate R were encoded using two independent multilayer perceptrons. Each encoder consisted of two fully connected layers with dimensions of 681→256→128. The 256-dimensional hidden layer was followed by ReLU activation and dropout with a rate of 0.3, and the final layer generated a 128-dimensional developmental-change feature vector. Therefore, the two branches produced $F_{\Delta s}$ and $F_{R}$, respectively, with the same dimensionality as the D2 and D5 stage-specific features. No parameters were shared between the two developmental-change encoders. Together, the two multilayer perceptrons contained 414,976 trainable parameters.

#### 2.4.4. Stage-Aware Attention Fusion Module

**Figure 5:**
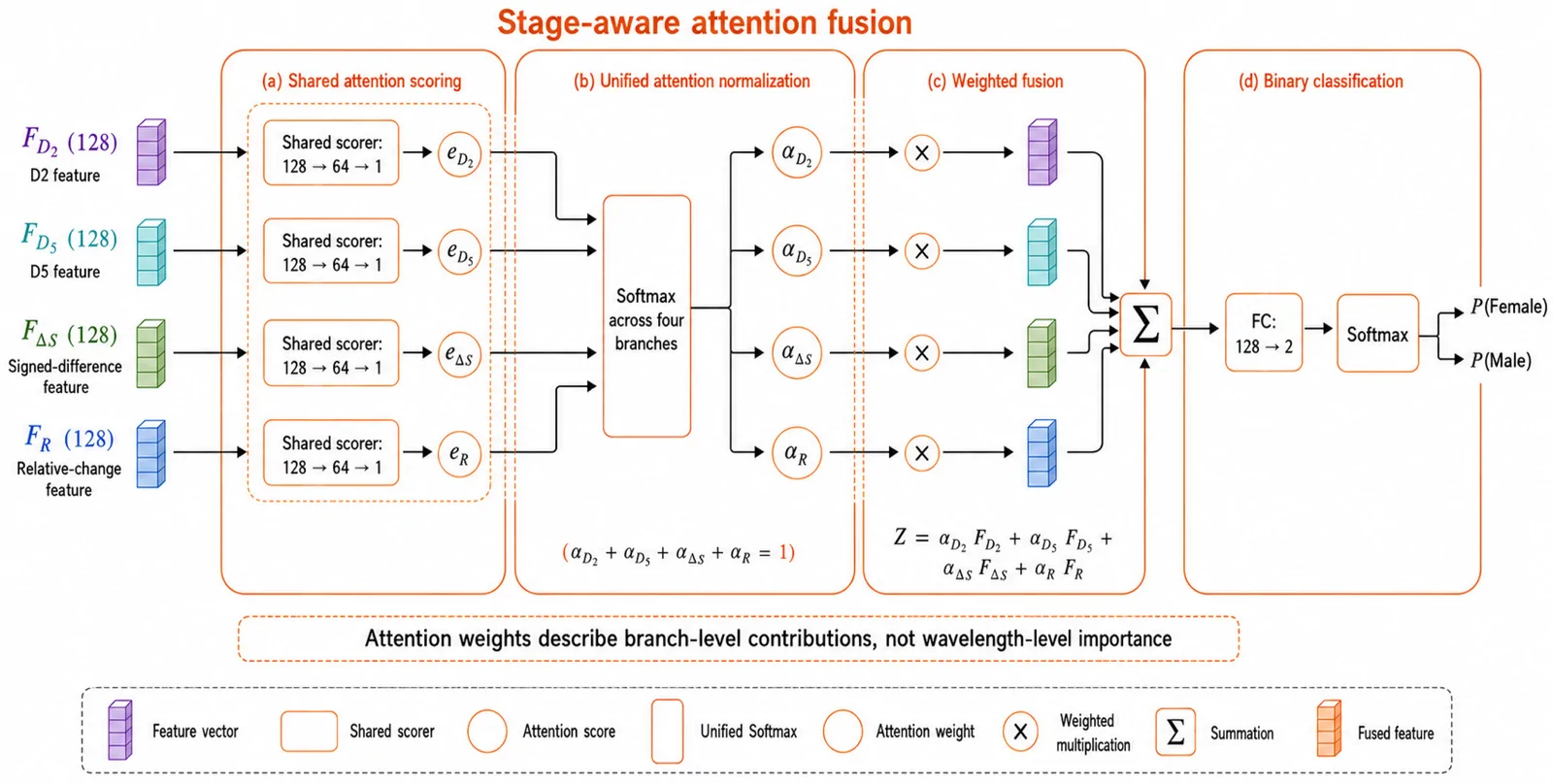
Architecture of the stage-aware attention fusion module.

The final DSSF-Net generated four 128-dimensional candidate feature vectors, corresponding to the D2 spectrum, D5 spectrum, signed difference spectrum ΔS, and relative change rate R. As shown in Figure 5, the four candidate features were evaluated using a shared attention scorer with dimensions of 128→64→1. The resulting four scalar scores were jointly normalized using a single Softmax function, ensuring that the attention weights across all four branches summed to one.

(10) $$e_{i,k}=v^{T}\tanh\left(Wq_{i,k}+b\right)$$

(11) $$\alpha_{i,k}=\frac{\exp\left(e_{i,k}\right)}{\sum_{I=1}^{K}\exp\left(e_{i,I}\right)}$$

(12) $$z_{i}=\overset{K}{\underset{k=1}{\sum }}\alpha_{i,k}q_{i,k}$$

(13) $${\hat{y}}_{i}=softmax\left(W_{o}z_{i}+b_{o}\right)$$

Here $q_{i,k}\;$ denotes the k-th candidate feature embedding of the i-th egg, and $\alpha_{i,k}$ denotes its attention weight normalized by the Softmax function. In the final DSSF-Net, K = 4, corresponding to the feature embeddings derived from the D2 spectrum, D5 spectrum, signed difference spectrum ΔS, and relative change rate R. The first expression computes the fused representation $z_{i}$ as the weighted sum of the four candidate feature embeddings, allowing the model to adaptively adjust their relative contributions for each egg [25]. In the second expression, $W_{o}$ and $b_{o}$ denote the weight matrix and bias vector of the final fully connected classification layer, respectively. The Softmax function converts the classification logits into the predicted class-probability vector ${\hat{y}}_{i}$, containing the probabilities that the i-th egg belongs to the female and male classes.

Compared with early fusion or simple concatenation, stage-aware attention can adaptively adjust contributions of different stages and change branches for each sample, thereby reducing interference from redundant spectral variables and noisy features.

The fused feature $z_{i}$ is further fed into a fully connected classification layer, and the Softmax function outputs the predicted probabilities of female and male embryos. During training, a cross-entropy loss function is used, and the Adam optimizer is employed for parameter updates [41]; batch normalization and residual connections are introduced into the network architecture to improve training stability [42,43]; early stopping is also applied based on the validation-set loss.

### 2.5. Experimental Setup and Evaluation Strategy

#### 2.5.1. Baseline Models

To systematically evaluate model performance, representative traditional spectral classifiers, ensemble learners, and deep learning models were selected as baselines, including PLS-DA, SVM, RF, XGBoost, 1D-CNN, BiLSTM, and Transformer Encoder. DSSF-Net was used as the proposed model [17,18,19,20,21,22,23,24,25,26,27,44,45,46]. To ensure a fair comparison, all models used the same input features determined during the exploratory screening stage. The same data partitions, preprocessing procedures, and evaluation metrics were applied across models.

PLS-DA establishes linear discriminant relationships between spectral variables and sex labels [17,46,47]; SVM evaluates nonlinear classification in high-dimensional spectral space [18,45]; RF and XGBoost verify ensemble performance in nonlinear feature combination [19,20]; 1D-CNN extracts local neighborhood band features [26]; BiLSTM models bidirectional dependencies along the wavelength sequence [24]; Transformer Encoder captures global long-range band associations [25]; deep belief networks have also been applied to early embryo image recognition [44]; and DSSF-Net fuses static spectra and dynamic changes from two developmental stages.

In dual-day screening experiments, all candidate combinations used the same SVM classifier and unified hyperparameter optimization workflow to reduce structural bias in day-combination comparison. After the optimal dual-day combination was determined, full comparisons were conducted with different traditional and deep learning models. Hyperparameters of PLS-DA, SVM, RF, and XGBoost were optimized by exhaustive grid search with stratified five-fold internal cross-validation using only the current outer training data. The candidate numbers of latent variables for PLS-DA were 2, 5, 10, 15, and 20. For the radial-basis-function SVM, the candidate values were $∁\in \left\{0.1,1,10,100\right\}$ and $\gamma \in \left\{0.001,0.01,0.1,1\right\}$. For RF, the candidate numbers of trees were 100, 200, 300, and 500, and the candidate maximum depths were 5, 10, 20, and unrestricted. For XGBoost, the candidate numbers of estimators were 100, 200, 300, and 500, the candidate maximum depths were 3, 5, 7, and 10, and the candidate learning rates were 0.01, 0.05, 0.1, and 0.2. Mean AUC across the five internal validation folds was used as the hyperparameter-selection criterion. All data partitions were performed at the egg-ID level, and the outer evaluation fold and the fixed internal hold-out test set were excluded from hyperparameter tuning.

#### 2.5.2. Dataset Partitioning, Model Training, and Implementation Details

This study adopted a two-stage model-development strategy and evaluated model performance at multiple levels using stratified five-fold cross-validation, a fixed internal hold-out test set, and cross-batch validation. All data partitions were performed at the egg-ID level. Spectra collected from the same egg on different incubation days, together with all derived features, were always assigned to the same data subset, thereby preventing information leakage caused by placing multi-day information from the same egg in both the training and evaluation sets.

Before model development, stratified sampling by incubation batch and sex was used to reserve 200 of the 1600 eligible eggs as a fixed internal hold-out test set, comprising 100 male and 100 female embryo eggs. Specifically, 25 male and 25 female embryo eggs were sampled from each batch to ensure balanced batch and sex distributions within the independent test set. This test set remained sealed throughout model development and was not used for incubation-day screening, feature selection, estimation of spectral-preprocessing parameters, hyperparameter optimization, early-stopping decisions, classification-threshold determination, or model selection. It was used only after the four model configurations and their training settings had been fixed to obtain confirmatory performance estimates. No model architecture, hyperparameter, preprocessing procedure, or classification threshold was modified according to the independent-test results. The remaining 1400 eggs constituted the model-development set, with 350 eggs from each batch.

The first stage involved exploratory screening and internal confirmation. The 1400 model-development samples were divided once into a training set, a validation set, and an internal evaluation set at a ratio of 7:2:1, containing 980, 280, and 140 eggs, respectively. The incubation-batch and sex distributions were kept as balanced as possible across the three subsets [48,49,50,51,52]. All candidate incubation-day and feature combinations were evaluated using the same SVM classifier, spectral-standardization method, hyperparameter search space, and optimization procedure; only the input day or feature combination was varied. The training set was used for model fitting, whereas the validation-set AUC was used to compare the relative discriminatory performance of the D1–D7 single-day schemes, all 21 dual-day combinations, and the different multi-day and developmental-change feature combinations. After D2 + D5, ΔS, and R had been fixed based on the validation results, the selected input scheme was evaluated once on the 140-egg internal evaluation set.

The second stage involved model comparison and final performance evaluation. After the D2 + D5, ΔS, and R input scheme had been fixed using the validation set and confirmed once using the internal evaluation set, all 1400 model-development samples were recombined. PLS-DA, SVM, RF, XGBoost, 1D-CNN, BiLSTM, Transformer Encoder, and DSSF-Net were then compared using a unified egg-level stratified five-fold cross-validation procedure. The data were divided at the egg-ID level into five mutually exclusive folds, each containing approximately 280 eggs, while maintaining the incubation-batch and sex distributions as evenly as possible. In each round, four folds comprising approximately 1120 eggs were used for model training, and the remaining fold was used as the outer evaluation set. This process was repeated until each fold had served once as the outer evaluation set. All models used exactly the same five-fold partitions.

In each round of the outer five-fold cross-validation, the hyperparameter grid search for PLS-DA, SVM, RF, and XGBoost was independently conducted using stratified five-fold internal cross-validation within the current outer training portion. The hyperparameter combination with the highest mean internal-validation AUC was selected, and the corresponding model was refitted using the complete outer training portion before evaluation on the held-out outer fold. For the deep-learning models, the current outer training data were further divided into internal training and validation subsets. The models were optimized using the cross-entropy loss function, and early stopping was implemented according to the loss on the internal validation subset. The outer evaluation fold was not used for hyperparameter selection, model selection, or early-stopping decisions. DSSF-Net generated Softmax probabilities for the female and male classes, and the class with the higher probability was assigned as the prediction. Equivalently, an egg was classified as male when the predicted probability of the male class was at least 0.5 and as female otherwise. This classification threshold of 0.5 was specified in advance and was not optimized or adjusted using either the outer evaluation folds or the fixed internal hold-out test set. After five-fold cross-validation and model comparison had been completed, the input representation, network architecture, hyperparameters, and training settings of DSSF-Net were fixed. The final model was then retrained using the complete 1400-egg development set and evaluated once on the previously reserved independent test set of 200 eggs. For the five-fold model comparison, the male-class probabilities predicted for each egg in its held-out outer fold were pooled after completion of all five evaluation rounds. Each egg therefore contributed exactly one out-of-fold prediction. After model development had been completed, the final DSSF-Net was retrained using the complete 1400-egg development set and evaluated once on the fixed internal hold-out test set of 200 eggs. The prespecified classification threshold of 0.5 was used separately to generate the confusion matrix and threshold-dependent metrics.

To confirm whether the additional D2 acquisition provided a reproducible predictive benefit on untouched data, four predefined configurations were evaluated on the same fixed 200-egg independent test set. The D5-only baseline used only the D5 spectrum with a single stage-specific spectral encoder and classification layer. The Direct D2 + D5 baseline directly concatenated the D2 and D5 spectral vectors before classification, without using the ΔS branch, R branch, or stage-aware attention. The D2 + D5 + ΔS + R configuration retained the four input branches but replaced the stage-aware attention module with unweighted averaging of the four branch embeddings before the final classifier. The complete DSSF-Net used the two stage-specific spectral branches, the ΔS and R developmental-change branches, and the stage-aware attention module described in Section 2.4.4. All four configurations were trained using the complete 1400-egg development set and evaluated using the same prespecified classification threshold of 0.5. No model architecture, hyperparameter, preprocessing procedure, or classification threshold was modified according to the independent-test results.

To further assess the generalization ability of the complete modeling procedure when applied to an unseen incubation batch, nested leave-one-batch-out validation was conducted in addition to the two-stage model-development procedure. Cross-batch validation used only the 1400 eggs in the model-development set. Because 50 eggs from each original batch had already been assigned to the fixed internal hold-out test set, each batch contributed 350 eggs to the model-development set. In each round, three batches comprising 1050 eggs were used for model development, and all 350 development-set eggs from the remaining batch were used as the external batch-level evaluation set. Four rounds were conducted so that each batch served once as the external evaluation batch.

To prevent batch-level information leakage, the external evaluation batch was completely withheld at the beginning of each cross-batch validation round. Incubation-day combination screening, developmental-change feature selection, estimation of spectral-preprocessing parameters, hyperparameter optimization, and determination of early-stopping conditions were performed using only the three training batches. The prespecified classification threshold of 0.5 was used in every round, and the external evaluation batch was not used for threshold selection, threshold adjustment, or probability calibration. The held-out batch did not participate in any part of model development and was evaluated only once after the model and its parameters had been finalized for that round. The purpose of cross-batch validation was not to use the evaluation batch to reselect the final incubation-day combination, but to determine whether the complete workflow—including incubation-day screening, feature construction, and model training performed exclusively on existing batches—could generalize to a previously unseen incubation batch.

To ensure a fair comparison, all models used the same input features determined during the exploratory screening stage, the same egg-level five-fold data partitions, and the same evaluation metrics. The selected model-specific settings for the conventional machine-learning models were as follows: PLS-DA used 10 latent variables; SVM used the radial basis function kernel with C = 10 and γ = 0.01; RF used 200 trees with a maximum depth of 10; and XGBoost used 200 estimators, a learning rate of 0.05, and a maximum depth of 5. For deep-learning models, the shared training settings are listed in Table 1, and the model-specific architectures and trainable parameter counts are summarized in Table 2.

Table 2 summarizes the model-specific architectures and trainable parameter counts used in the deep-learning experiments. To ensure consistency in input information, all models used the same four input features and produced a 128-dimensional representation before binary classification. The 1D-CNN, BiLSTM, and Transformer Encoder contained 36,642, 135,426, and 265,858 trainable parameters, respectively. DSSF-Net contained 515,971 trainable parameters because it employed two independent stage-specific convolutional encoders, two developmental-change multilayer perceptron encoders, and a stage-aware attention module. Thus, DSSF-Net had a higher model capacity than the comparator networks. Because a parameter-matched control model was not included, the subsequent performance differences cannot completely isolate the effects of the proposed feature representations and fusion architecture from those of increased model capacity.

#### 2.5.3. Characteristic Wavelength Selection and Model Interpretability Analysis

**Figure 6:**
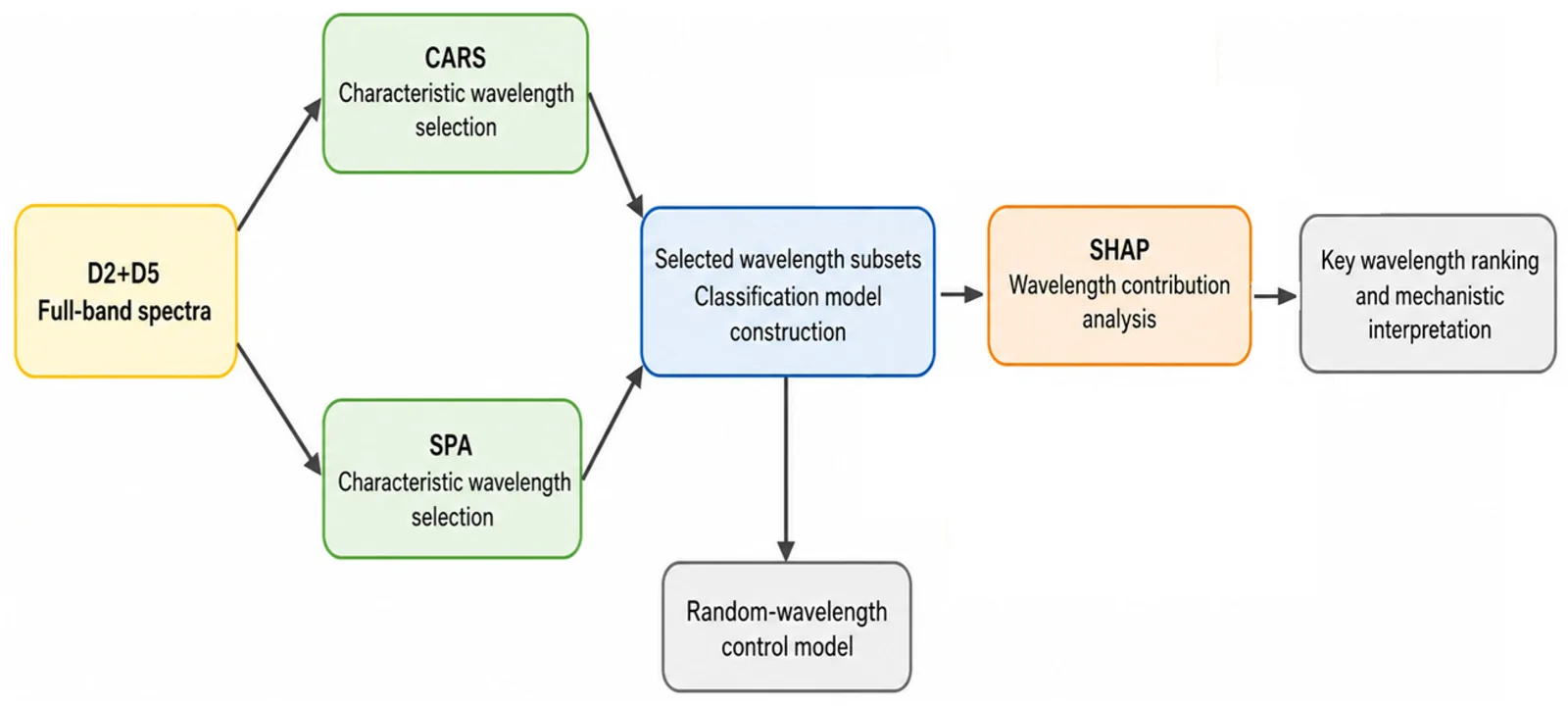
Workflow of characteristic wavelength screening and model interpretability analysis.

After the key incubation days, developmental-change features, model architecture, and evaluation procedure had been determined, a post-hoc wavelength-level interpretation analysis was conducted for the final DSSF-Net, as illustrated in Figure 6. This analysis aimed to identify candidate spectral regions that contributed strongly to model predictions, while the subsequently developed reduced-band models were used solely to evaluate the predictive value of these candidate wavelength regions.

To evaluate the predictive value of the candidate spectral regions and the agreement among different interpretation methods, nested wavelength selection, random-wavelength controls, reduced-band model validation, and post-hoc interpretation analyses were conducted. These analyses focused on whether the selected wavelengths could retain the principal discriminatory performance using fewer variables and whether different methods identified similar candidate spectral regions.

All additional analyses employed egg-level stratified five-fold cross-validation. In each round, one outer evaluation fold was withheld, while spectral standardization, wavelength selection, hyperparameter optimization, and model training were performed exclusively using the remaining four folds to prevent information leakage. The D2 and D5 spectra and their corresponding ΔS and R features from the same egg were always assigned to the same data subset. Wavelength selection was performed using competitive adaptive reweighted sampling (CARS) and the successive projections algorithm (SPA). In each round, a full-band model, CARS- and SPA-based reduced-band models, and random-control models retaining the same numbers of wavelengths were constructed. Random wavelength selection was repeated 20 times within each training fold to determine whether performance differences were attributable to the predictive value of the selected wavelengths rather than merely to dimensionality reduction. All models used the same DSSF-Net architecture, training procedure, and evaluation metrics, with only the input-layer dimension being adjusted.

Post-hoc wavelength-level interpretation was performed using SHAP and local feature perturbation. Local perturbation was conducted using spectral windows of approximately 3 nm. The features within each target window were replaced with their corresponding training-fold means, and wavelength importance was quantified by the reduction in outer-fold evaluation AUC after perturbation. Attention weights were used only to characterize the relative contributions of the D2, D5, ΔS, and R branches and were not treated as wavelength-level explanatory evidence.

Regions consistently identified by CARS, SPA, SHAP, and local perturbation were regarded as candidate spectral intervals with relatively high predictive value. Because the spectra were sampled at 1 nm intervals, whereas the actual spectral resolution of the instrument was approximately 2.8 nm, the results were interpreted in terms of continuous spectral regions rather than individual wavelength points. The cross-batch stability and biological relevance of these regions require further validation using additional independent datasets.

#### 2.5.4. Evaluation Metrics

Accuracy, precision, recall, F1-score, sensitivity, specificity, and area under the receiver operating characteristic curve (AUC) were used as primary metrics. Confusion matrices and ROC curves were also used to visualize classification performance for male and female embryos.

In all binary classification analyses, male embryonated eggs were defined as the positive class, whereas female embryonated eggs were defined as the negative class. Accordingly, TP, TN, FP, and FN denote correctly classified male eggs, correctly classified female eggs, female eggs misclassified as male, and male eggs misclassified as female, respectively. The main evaluation metrics were calculated as follows:

(14) $$\mathrm{Accuracy}\;=\;\frac{\mathrm{TP}\;+\;\mathrm{TN}}{\mathrm{TP}\;+\;\mathrm{TN}\;+\;\mathrm{FP}\;+\;\mathrm{FN}}$$

(15) $$\mathrm{Precision}\;=\frac{\mathrm{TP}}{\mathrm{TP}\;+\;\mathrm{FP}}$$

(16) $$\mathrm{Recall}\;=\frac{\mathrm{TP}}{\mathrm{TP}\;+\;\mathrm{FN}}$$

(17) $$\mathrm{Specificity}\;=\frac{\mathrm{TN}}{\mathrm{TN}\;+\;\mathrm{FP}}$$

(18) $$F1\text{-}\mathrm{score}\;=\frac{2\mathrm{PR}}{P\;+\;R}$$

For five-fold cross-validation and four-round leave-one-batch-out validation, performance metrics were reported as mean ± standard deviation across the corresponding evaluation folds or held-out batches.

Related literature on deep model interpretation, egg transmission spectroscopy and sex identification, NIR fundamentals, and precision livestock farming provided background for experimental design and result comparison [53,54,55,56,57,58,59].

For the fixed internal hold-out test set, two-sided 95% confidence intervals for accuracy, sensitivity, specificity, positive predictive value, and negative predictive value were calculated using the Wilson score method, with each egg treated as one independent analysis unit. The 95% confidence interval for the AUC was estimated using 2000 egg-level class-stratified bootstrap resamples of the fixed sample-level label–probability pairs. In each bootstrap resample, male and female eggs were sampled separately with replacement to preserve the original class sizes of 100 male and 100 female eggs. The AUC was recalculated for each resample, and the 2.5th and 97.5th percentiles of the bootstrap AUC distribution were used as the lower and upper confidence limits, respectively. The model was not retrained, and no model parameter or classification threshold was adjusted during bootstrap resampling. Mean ± standard deviation was reported only for the five-fold cross-validation and four-round leave-one-batch-out analyses, whereas the fixed internal hold-out results were reported as point estimates with 95% confidence intervals.

## 3. Results and Discussion

This section follows the logic of spectral response patterns, key incubation-day screening, dynamic feature construction, model validation, and interpretability analysis. Transmission spectral changes during D1–D7 are first analyzed to clarify stage-dependent sex-related information. Key complementary day combinations and cross-day developmental-change features are then screened. Finally, cross-batch validation, model comparison, ablation experiments, and wavelength-level interpretation are used to evaluate effectiveness and application potential in smart hatchery scenarios.

### 3.1. Spectral Dynamics and Sex-Related Optical Differences During Early Incubation

**Figure 7:**
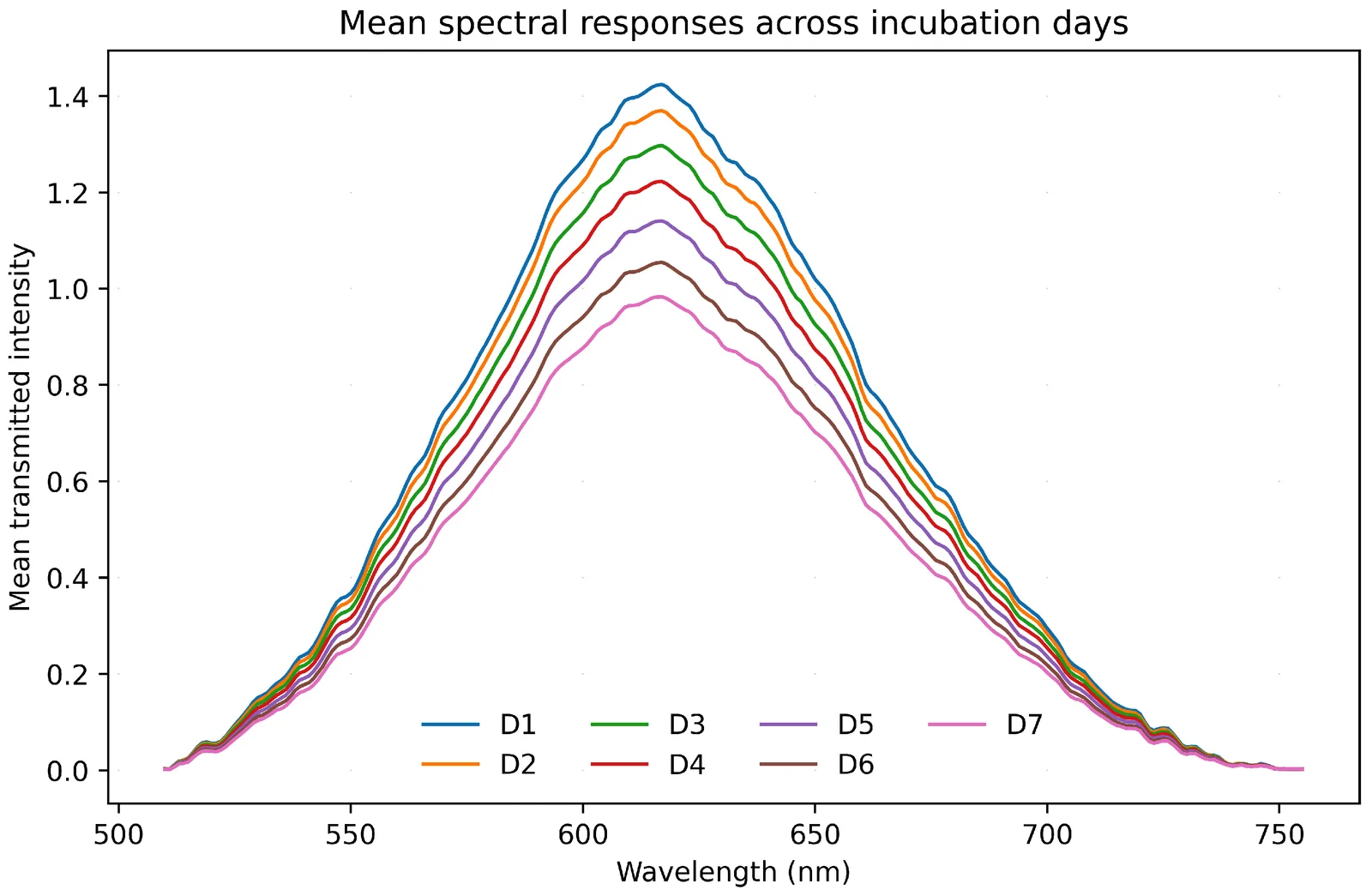
Mean transmission spectral response curves of eggs at different incubation days.

Within the broader spectral interval of 510–755 nm, where post-hoc model-derived importance was relatively concentrated, egg transmission spectra showed stable responses that reflected optical changes during early embryonic development. As shown in Figure 7, mean spectral curves across incubation days had similar overall shapes, with main peaks in the middle visible region, but transmission intensity decreased continuously with incubation. For male embryos, mean transmission intensity within the displayed 510–755 nm interval decreased from 0.603 on D1 to 0.417 on D7; female embryos showed the same trend. This indicates that early development mainly alters light absorption and transmission through changes in internal tissue, vasculature, and scattering structures rather than changing the overall spectral shape.

**Figure 8:**
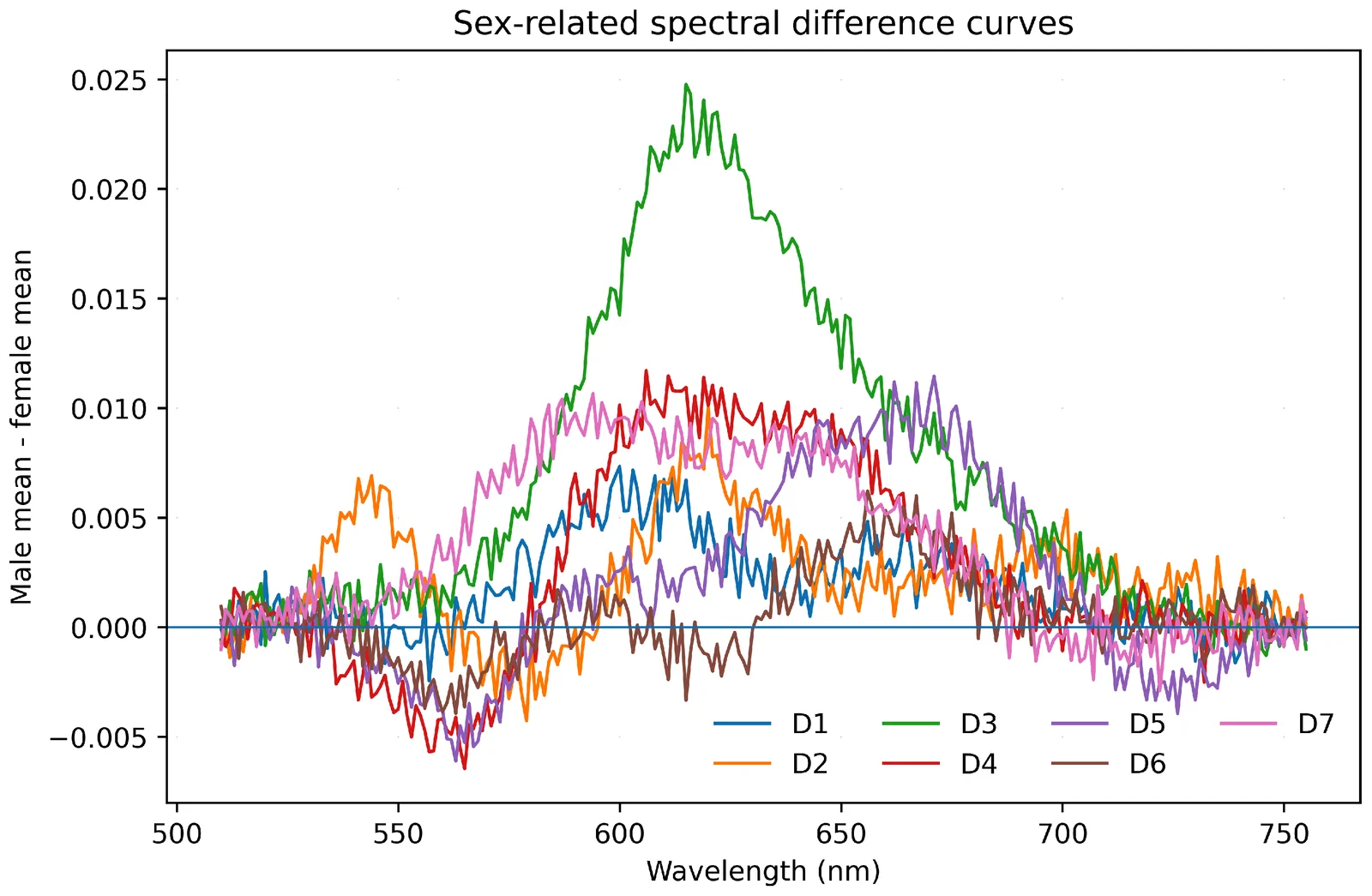
Sex-related spectral difference curves between male and female embryos during early incubation.

To locate sex-related responses, mean spectral differences between male and female embryos were calculated for each day. As shown in Figure 8, sex differences were not uniformly distributed across all days and wavelengths, but showed clear stage dependence and band selectivity. D3 had the highest mean absolute difference of 0.0072, with a maximum absolute difference of 0.0248 at 615 nm. D1 and D6 had mean absolute differences of only 0.0021 and 0.0015, respectively, indicating weak sex contrasts. Local difference peaks also appeared near 620 nm (D2), 671 nm (D5), and 594 nm (D7).

It should be noted that the relatively high peaks and mean signed difference spectrum observed for D3 in the raw difference curves only reflect the overall distance between the population-averaged spectra of male and female embryos, and do not directly represent classification ability at the individual-egg level. Actual classification performance is also affected by within-class variation, overlap between the two class distributions, measurement noise, band redundancy, and individual differences. Therefore, subsequent classification experiments are required to determine whether each incubation day provides stable individual-level discriminative information and whether complementary information exists among different days.

### 3.2. Key Incubation-Day Selection and Developmental-Change Feature Evaluation

Based on the above spectral differences, the modeling value of different incubation days was further evaluated. Single-day models for D1–D7 were first constructed to assess the independent discriminative ability of each time point. All dual-day and multi-day combinations were then compared to identify key time windows with high performance.

**Figure 9:**
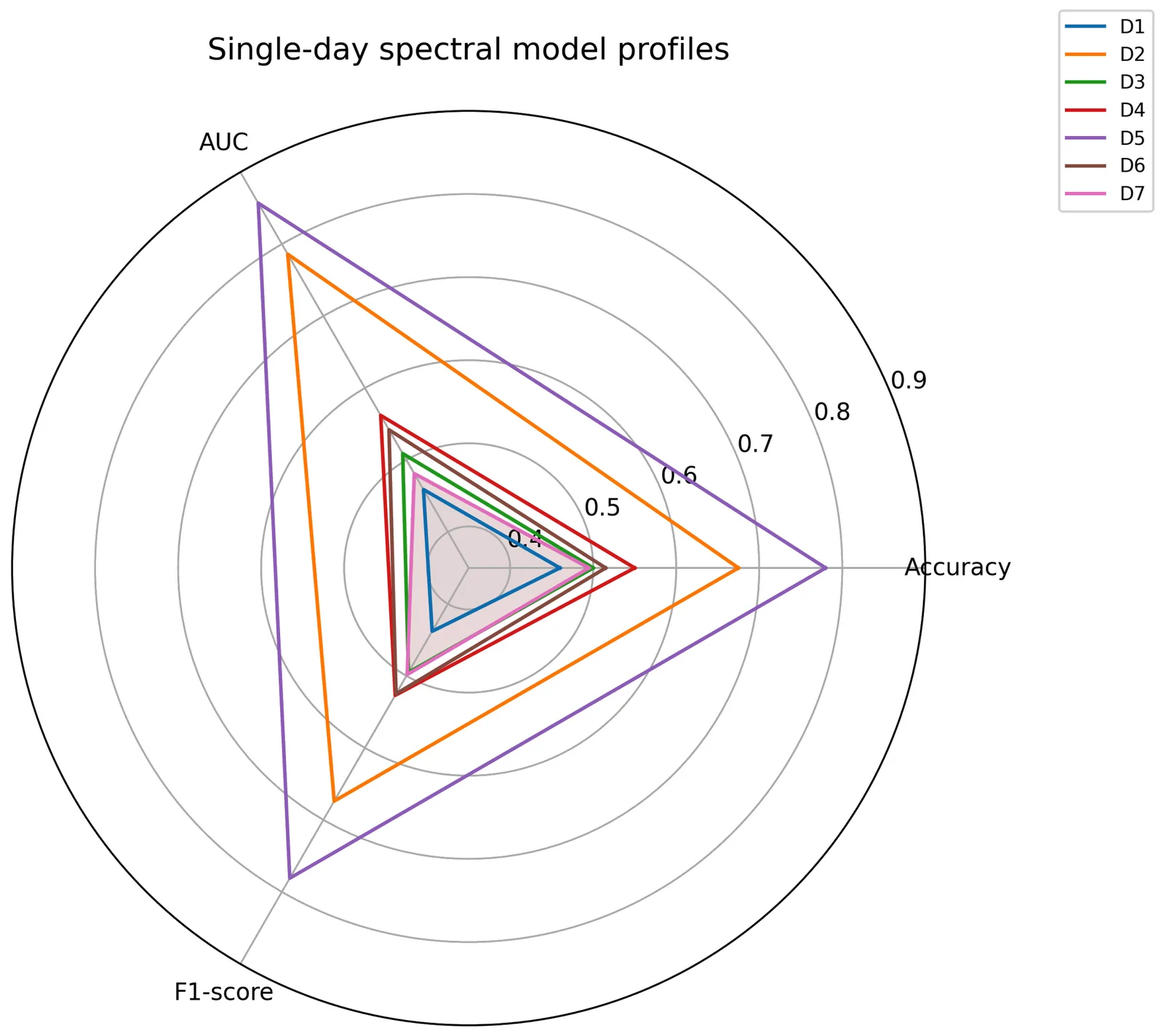
Exploratory validation performance of the single-day SVM models under the fixed data partition.

As shown in Figure 9, a radar chart displays multi-metric performance of single-day models, with radial axes for accuracy, AUC, and F1-score. Larger enclosed areas indicate stronger overall discrimination. D5 was the best single-day window, with accuracy, AUC, and F1-score of 0.780, 0.857, and 0.781, respectively. D2 ranked second (0.675, 0.786, and 0.674). Under the fixed exploratory validation split, D1, D3, D6, and D7 showed lower numerical discrimination than D2 and D5. D4 showed intermediate numerical performance, higher than the lowest-performing days but lower than D2 and D5.

Single-day results suggested that D2 and D5 may represent two different but valuable developmental stages. D2 is closer to early embryonic development and may capture earlier sex-related optical differences, whereas D5 may contain more pronounced tissue development, vascular changes, and metabolic responses. All 21 dual-day combinations within D1–D7 were therefore compared.

Although D3 exhibited a relatively large population-level mean spectral difference between sexes, its numerical discriminatory performance under the fixed exploratory split was lower than that of D2 and D5. This may be because the between-sex differences observed on D3 were accompanied by substantial within-class variation and overlap between the male and female sample distributions. Alternatively, some of these differences may have reflected overall spectral shifts associated with eggshell translucency, initial transmission intensity, and early embryonic developmental status.

**Figure 10:**
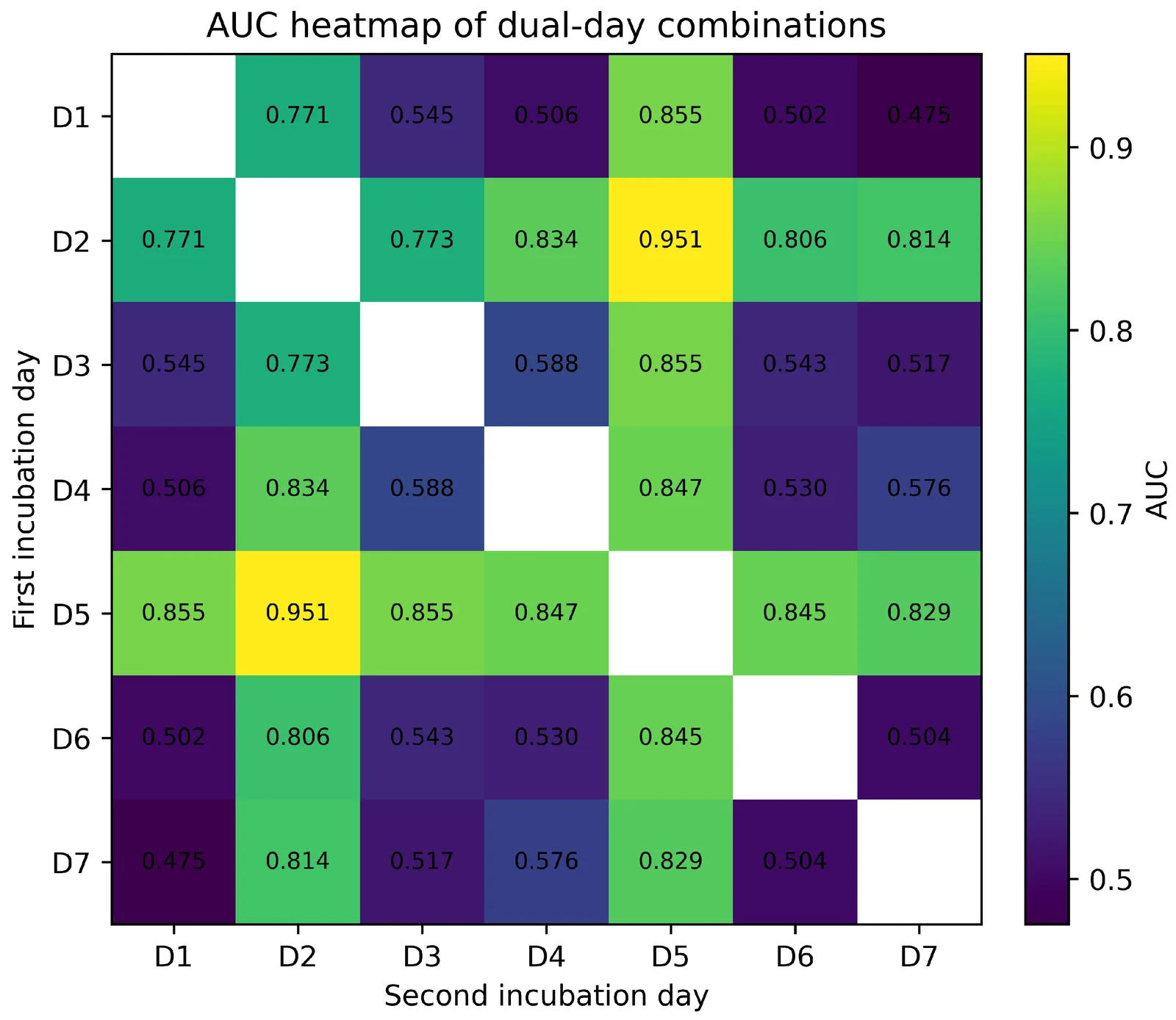
Exploratory validation AUC heatmap of the 21 dual-day spectral combinations using the same SVM classifier and data partition.

As shown in Figure 10, AUC values varied substantially among dual-day combinations in the heatmap. Darker colors indicate higher AUC, and the blank diagonal represents same-day combinations excluded from dual-day analysis. D2 + D5 achieved the highest AUC of 0.951, with accuracy and F1-score of 0.885 and 0.886, respectively. D3 + D5 and D1 + D5 both reached AUC of 0.855, 0.096 lower than D2 + D5, indicating stronger complementarity between D2 and D5 than other D5-containing pairs.

To directly evaluate the incremental value of adding a D2 measurement, D5-only and D2 + D5 were compared under the same exploratory screening conditions using identical egg-level data partitions, SVM classifiers, and hyperparameter optimization procedures. Adding the D2 measurement increased accuracy, validation AUC, and F1-score by 0.105, 0.094, and 0.105, respectively. These results indicate that although D2 alone provided limited discriminatory performance, it contributed complementary early developmental information that improved the reliability of discrimination based on D5 spectra. Because both schemes produced the final decision on D5, D2 + D5 offered no advantage in terms of decision timing. Its primary benefit was improved discriminatory performance at the cost of one additional measurement and longitudinal egg tracking.

To determine whether more sampling days were needed, fusion schemes with 2–7 days were compared. As shown in Table 3, D2 + D5 achieved the highest AUC. When three days were fused, D2 + D5 + D6 reached AUC of 0.950, comparable to D2 + D5, but accuracy and F1-score did not improve further. When all D1–D7 spectra were used, AUC decreased to 0.905. These results indicate that the key to multi-day fusion is not increasing input quantity, but whether newly added days provide effective and complementary information.

**Figure 11:**
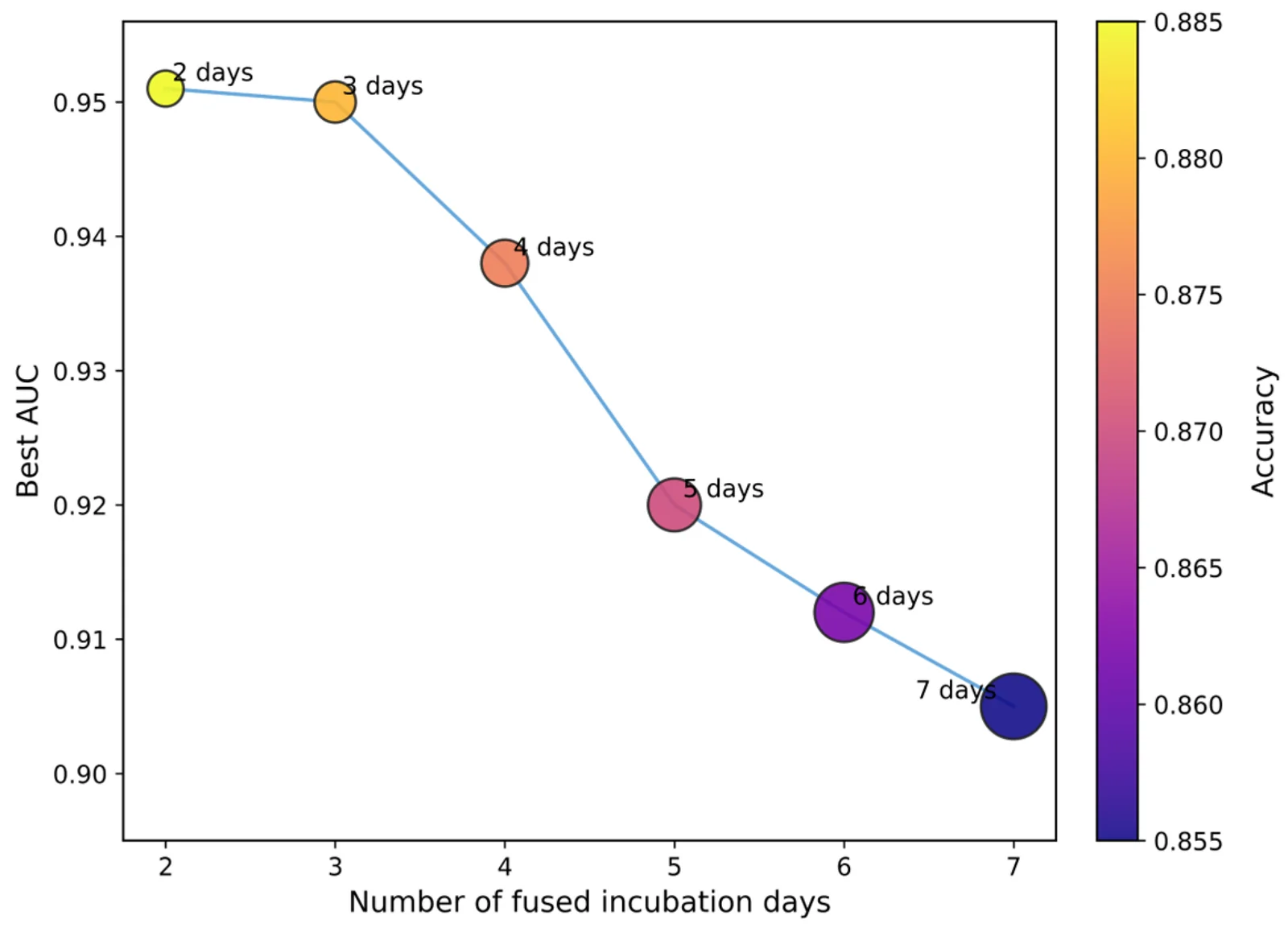
Exploratory comparison of validation AUC among spectral combinations with different numbers of acquisition days.

As shown in Figure 11, the bubble chart summarizes the relationship between classification performance and the number of spectral acquisition days. The x-axis represents the number of fused incubation days, the y-axis represents the best AUC, and bubble color indicates accuracy. Bubble size is scaled according to the number of spectral acquisition days and is used only to visualize the complexity of the longitudinal sampling scheme rather than economic or operational cost. As the number of included days increased from two to seven, the best AUC did not improve consistently and instead showed an overall declining trend. Among the evaluated combinations, D2 + D5 achieved high discrimination performance using two complementary measurement days. Therefore, D2 and D5 were selected as the key early measurement windows for subsequent modeling, providing a basis for simplifying the longitudinal acquisition scheme without implying a quantified commercial cost advantage.

After identifying D2 + D5 as the core combination, cross-day developmental-change features were constructed. Simple concatenation of D2 and D5 raw spectra uses only static information from two stages and cannot directly express change direction and magnitude. After identifying D2 + D5 as the core combination, the signed difference spectrum ΔS and relative change rate R were calculated according to Equations (8) and (9).

**Figure 12:**
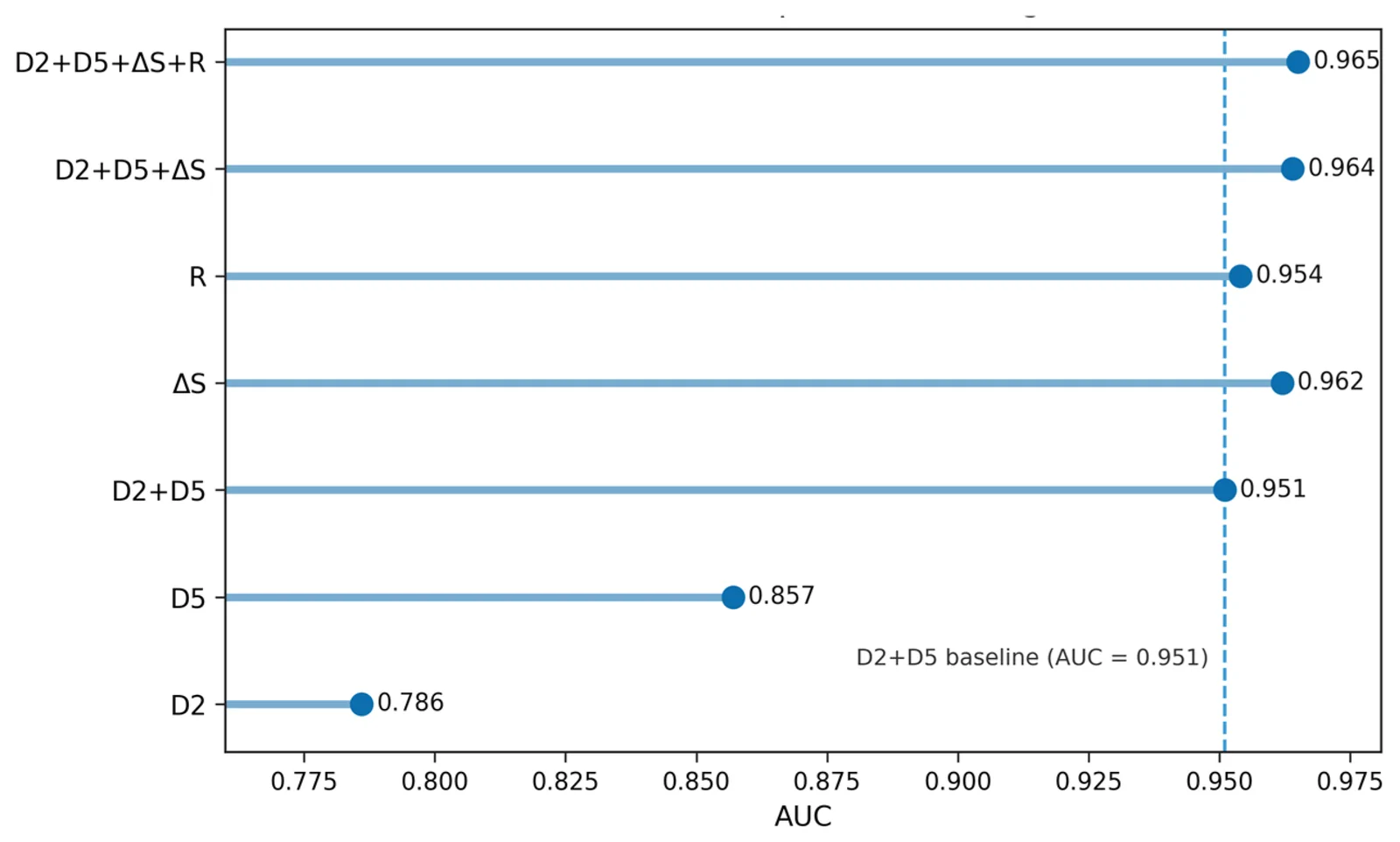
Exploratory validation AUC of different developmental-change feature combinations based on D2 and D5.

As shown in Figure 12, a lollipop chart presents AUC gains from different feature sets. Using ΔS alone achieved AUC of 0.962, higher than 0.951 for simple D2 + D5 concatenation. Using R alone yielded accuracy and F1-score of 0.895 and 0.896. When D2, D5, ΔS, and R were fused, AUC further reached 0.965.

These results indicate that sex-related information exists not only in static spectra at individual days, but also in dynamic change trajectories between developmental stages. ΔS preserves both the direction and magnitude of wavelength, whereas R reflects relative change trends and can partially reduce effects of individual initial transmission intensity. Compared with blindly increasing sampling days, constructing developmental-change features around D2 and D5 is more consistent with model simplification and production deployment needs in non-destructive detection.

### 3.3. Cross-Batch Validation

**Figure 13:**
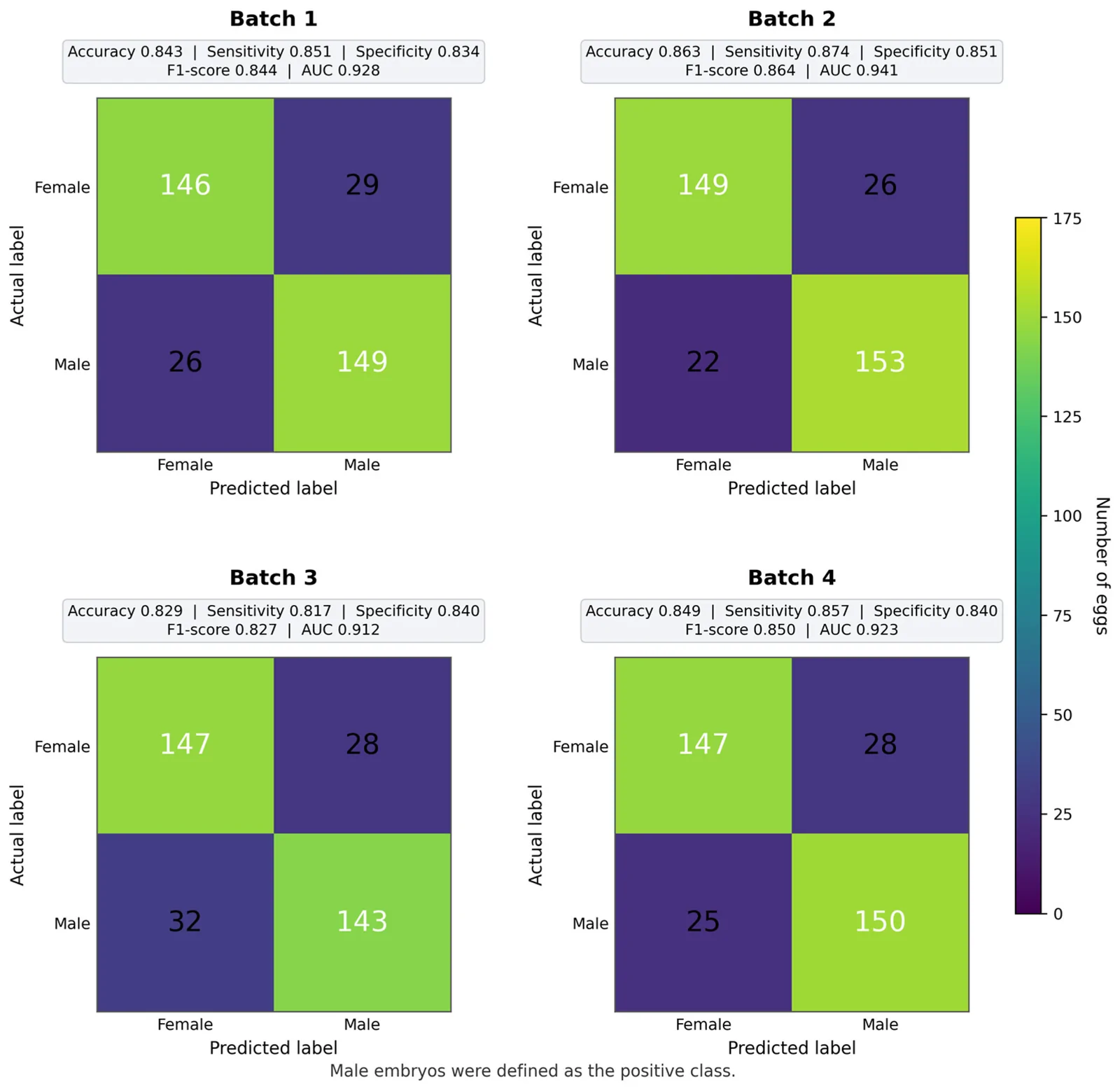
Cross-batch validation performance.

The confusion matrices obtained from the four rounds of nested leave-one-batch-out validation are shown in Figure 13. In each round, male embryo eggs were designated as the positive class, and the model was evaluated on an external batch containing 175 female and 175 male embryo eggs.

When Batch 1 served as the external evaluation set, the model correctly classified 146 female and 149 male embryo eggs, while 29 female eggs were misclassified as male and 26 male eggs were misclassified as female. The corresponding accuracy, sensitivity, and specificity were 0.843, 0.851, and 0.834, respectively. When Batch 2 was used as the external evaluation set, the model correctly classified 149 female and 153 male embryo eggs, with only 26 female and 22 male eggs being misclassified. The resulting accuracy was 0.863, the highest among the four validation rounds.

For Batch 3, the model correctly classified 147 female and 143 male embryo eggs, while 32 male eggs were misclassified as female. The relatively high number of false negatives reduced sensitivity to 0.817 and accuracy to 0.829. For Batch 4, the model correctly classified 147 female and 150 male embryo eggs, with 28 female and 25 male eggs being misclassified. The corresponding accuracy, sensitivity, and specificity were 0.849, 0.857, and 0.840, respectively.

Across the four external batches, the model correctly classified a total of 589 female and 595 male embryo eggs. The mean accuracy, sensitivity, and specificity were 0.846 ± 0.014, 0.850 ± 0.024, and 0.841 ± 0.007, respectively. The numbers of false positives ranged from 26 to 29, whereas the numbers of false negatives ranged from 22 to 32. The two error types were generally comparable, with no clear or persistent prediction bias toward either class. Batch 2 yielded the best overall classification results, whereas Batch 3 showed a relatively high number of missed male embryo eggs, indicating that differences in data distribution among incubation batches can affect model performance under a fixed classification threshold. Nevertheless, the AUC exceeded 0.90 for all four external batches, demonstrating that the model retained good sample-ranking ability. Overall, DSSF-Net maintained relatively stable classification performance across the four unseen batches, further supporting its cross-batch generalization potential.

### 3.4. Model Comparison Based on Stratified Five-Fold Cross-Validation

After D2 + D5, the signed spectral difference (ΔS), and the relative change rate (R) had been determined during exploratory screening, different classification models were compared using a unified five-fold cross-validation procedure to further examine whether the performance improvement was primarily attributable to feature construction or model architecture. All models used the same features derived from D2, D5, ΔS, and R as inputs and were evaluated using identical data folds and performance metrics.

As shown in Table 4, PLS-DA achieved the lowest performance (accuracy 0.755, AUC 0.827, F1-score 0.754), indicating that linear discrimination is insufficient for complex nonlinear spectral changes during incubation. SVM, RF, and XGBoost improved performance, with XGBoost reaching AUC of 0.916. The deep learning models generally achieved numerically higher AUC values than the traditional models. The AUC values of 1D-CNN, BiLSTM, and Transformer Encoder were 0.931, 0.941, and 0.956, respectively.

**Figure 14:**
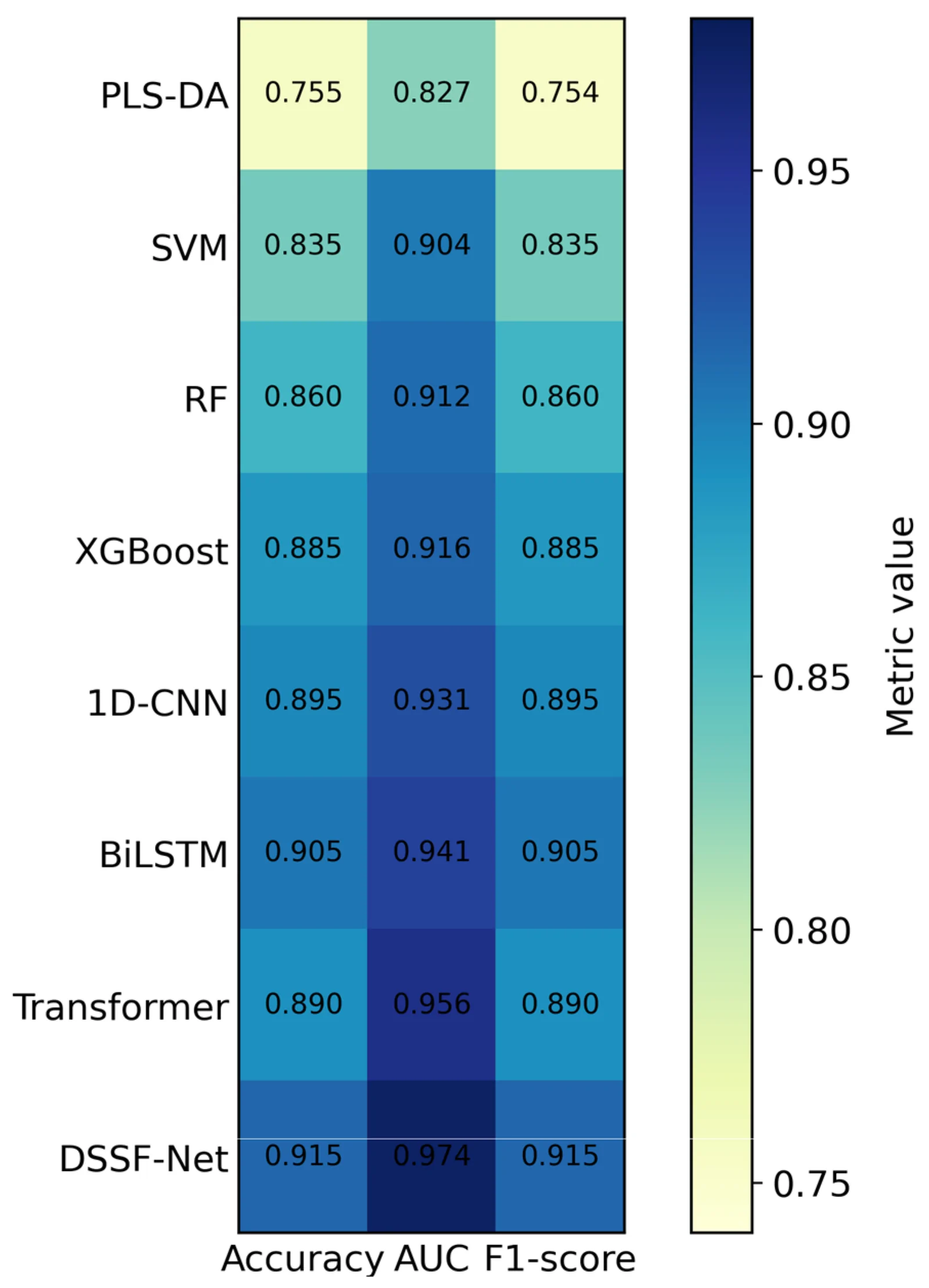
Heatmap of classification performance across different models.

As shown in Figure 14, a performance heatmap integrates accuracy, AUC, and F1-score across models. Darker colors indicate higher values. The heatmap shows numerical differences in accuracy, AUC, and F1-score among the evaluated models. DSSF-Net achieved relatively high values across all three metrics, rather than showing an increase in only one evaluation metric.

DSSF-Net achieved the highest numerical values among the evaluated models, with an accuracy of 0.915, an AUC of 0.974, and an F1-score of 0.915. Among the baseline models, BiLSTM achieved the highest accuracy and F1-score, both reaching 0.905, whereas Transformer Encoder achieved the highest baseline AUC of 0.956. Therefore, compared with the strongest baseline for each corresponding metric, DSSF-Net showed numerical increases of 0.010 in accuracy and F1-score and 0.018 in AUC. These results indicate that the integration of D2 and D5 spectra with ΔS and R produced consistently high numerical values across the three-evaluation metrics.

**Figure 15:**
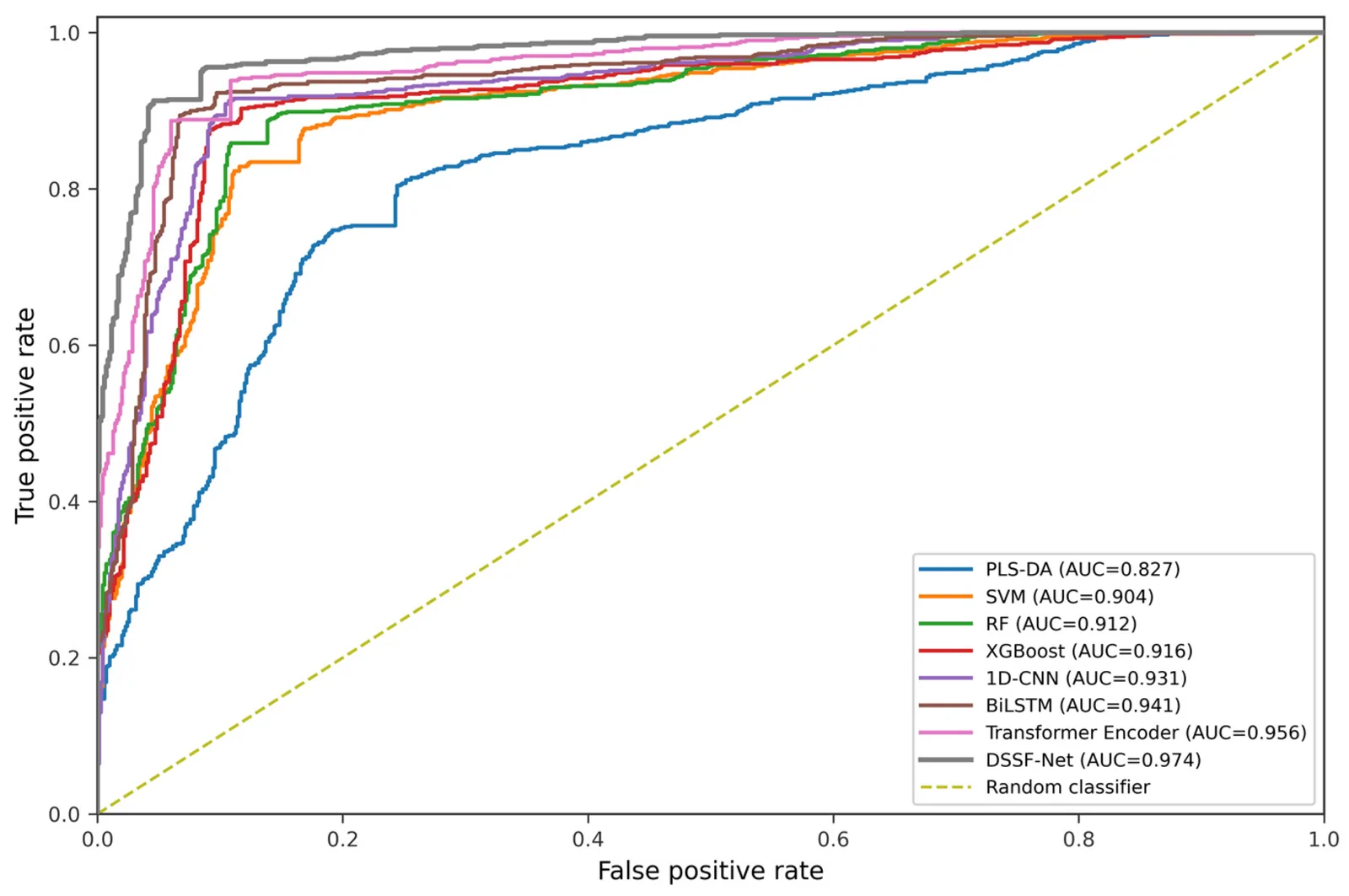
Pooled out-of-fold ROC curves of the evaluated models under unified egg-level stratified five-fold cross-validation.

Figure 15 presents the empirical ROC curves calculated directly from the sample-level probabilities predicted for the male class in the five held-out evaluation folds. Each egg contributed exactly one out-of-fold prediction, and the predictions were pooled only after all five-fold-wise evaluations had been completed. DSSF-Net achieved a mean fold-wise AUC of 0.974 ± 0.012 and showed favorable sample-ranking performance under the current five-fold cross-validation setting.

**Figure 16:**
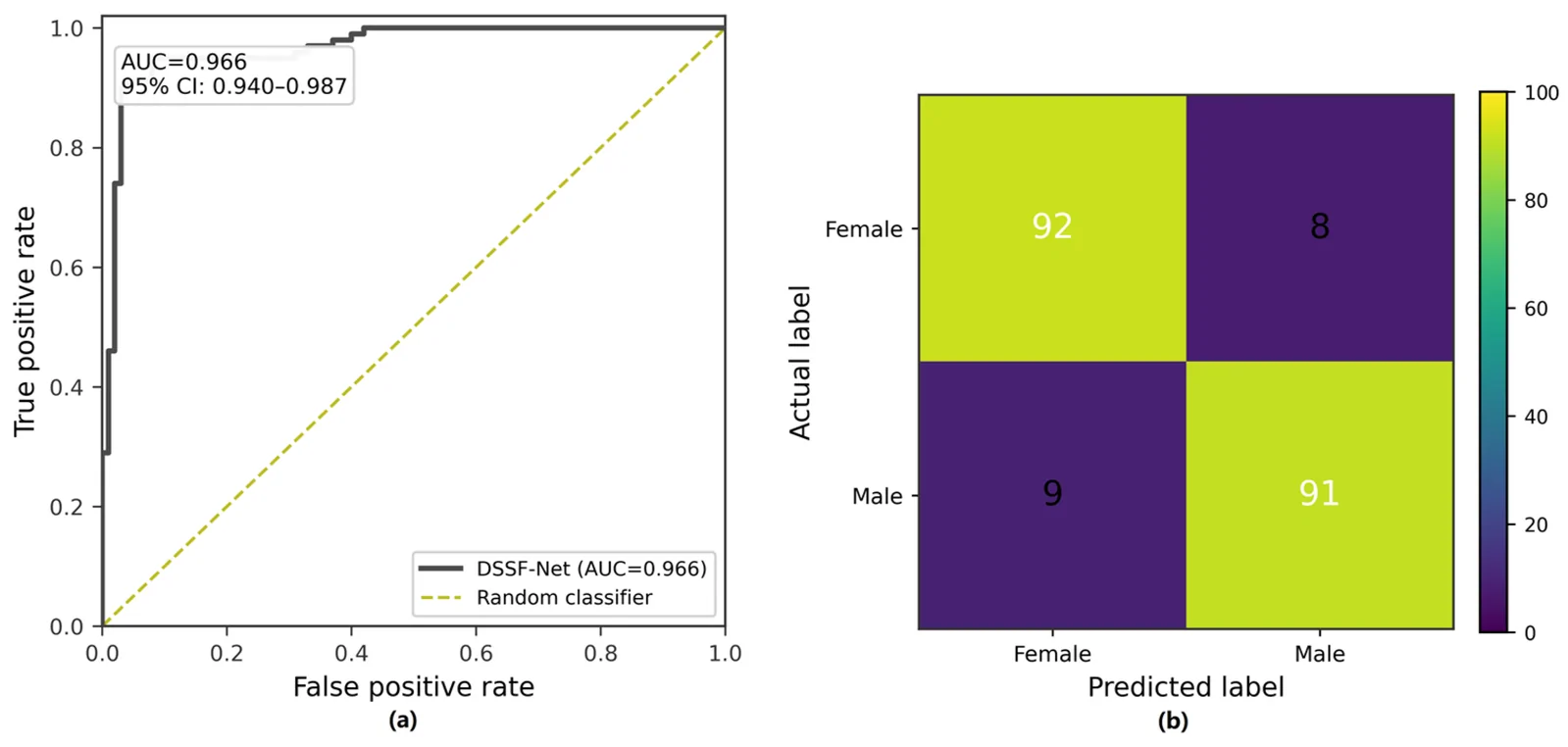
Performance of the final DSSF-Net on the fixed internal hold-out test set of 200 eggs: (**a**) empirical ROC curve, AUC = 0.966 (95% CI: 0.940–0.987); (**b**) confusion matrix at the prespecified threshold of 0.5, accuracy = 0.915 (95% CI: 0.868–0.946).

After the model architecture, input features, hyperparameters, and classification threshold had been fixed, the final DSSF-Net was retrained using the complete 1400-egg development dataset and evaluated once on the fixed 200-egg independent hold-out test set. The test set contained 100 male and 100 female embryos and was not involved in incubation-day screening, feature selection, hyperparameter optimization, early-stopping decisions, or classification-threshold determination. As shown in Figure 16, 92 female and 91 male embryos were correctly classified, whereas 8 female embryos were misclassified as male and 9 male embryos were misclassified as female. This resulted in an overall accuracy of 0.915 (95% CI: 0.868–0.946), with the confidence interval calculated using the Wilson score method and each egg treated as one analysis unit. The empirical AUC calculated directly from the sample-level male-class prediction probabilities of the 200 independent-test eggs was 0.966 (95% CI: 0.940–0.987). The ROC curve was generated directly from these sample-level probabilities and was not reconstructed or smoothed from summary AUC values. With male embryos defined as the positive class and the prespecified classification threshold fixed at 0.5, the sensitivity, specificity, positive predictive value, and negative predictive value were 0.910 (95% CI: 0.838–0.952), 0.920 (95% CI: 0.850–0.959), 0.919 (95% CI: 0.849–0.958), and 0.911 (95% CI: 0.839–0.952), respectively.

To determine whether the practical benefit of the additional D2 measurement could be reproduced on data that did not participate in incubation-day combination screening, the four predefined configurations were evaluated on the fixed internal hold-out test set. As shown in Table 5, the D5-only baseline achieved an accuracy of 0.790, an AUC of 0.865 (95% CI: 0.815–0.908), and an F1-score of 0.788. Under the same internal hold-out evaluation conditions, Direct D2 + D5 achieved an accuracy of 0.875, an AUC of 0.943 (95% CI: 0.909–0.969), and an F1-score of 0.876, corresponding to numerical increases of 0.085, 0.078, and 0.088, respectively, over D5-only.

After the ΔS and R branches were incorporated using non-attentive fusion, the accuracy, AUC, and F1-score increased to 0.905, 0.960 (95% CI: 0.932–0.980), and 0.905, respectively. The complete DSSF-Net with stage-aware attention achieved an accuracy of 0.915, an AUC of 0.966 (95% CI: 0.940–0.987), and an F1-score of 0.915. These results indicate that the additional D2 acquisition provided a numerical improvement over D5-only on the fixed internal hold-out test set, while the developmental-change features and stage-aware fusion provided further incremental gains. Because all four configurations produced their final decisions on D5, the additional D2 measurement improved predictive performance but did not advance the decision time.

Taken together, Table 4 and Figure 15 summarize model performance under the unified five-fold cross-validation procedure, whereas Figure 16 and Table 5 report performance on the fixed internal hold-out test set. On the independent test set, Direct D2 + D5 produced numerically higher accuracy, AUC, and F1-score than D5-only, providing confirmatory evidence that the D2 spectrum contained predictive information complementary to that of D5 on data that did not participate in day-pair selection. The further numerical improvements observed after incorporating ΔS, R, and stage-aware attention were consistent with incremental contributions from longitudinal developmental-change modeling and staged fusion, although the possible effect of increased model capacity cannot be completely excluded. Because all configurations generated their final predictions on D5, the additional D2 acquisition improved predictive performance rather than decision timing and required an additional measurement and longitudinal egg tracking.

### 3.5. Ablation Analysis of DSSF-Net Components

To quantify the incremental contributions of the major DSSF-Net components, sequential ablation experiments were conducted using the same egg-level stratified five-fold cross-validation protocol as that used for the final model comparison. All configurations used identical data folds, preprocessing procedures, optimization settings, and early-stopping criteria, and the same prespecified decision threshold of 0.5. The baseline model directly concatenated the raw D2 and D5 spectra. The dual-stream spectral encoder, difference-spectrum branch, relative-change-rate branch, and stage-aware attention module were then sequentially introduced, with only one new component added at each step. The results are reported as the mean ± standard deviation across the five held-out folds.

As shown in Table 6, direct concatenation of the D2 and D5 spectra achieved an accuracy of 0.884 ± 0.021, an AUC of 0.951 ± 0.017, and a precision of 0.885 ± 0.020. Introducing the dual-stream encoder increased these values to 0.891 ± 0.019, 0.958 ± 0.015, and 0.893 ± 0.018, respectively, indicating that separate encoding preserved stage-specific information more effectively than direct concatenation. Adding the ΔS branch produced the largest single-step AUC increase, from 0.958 to 0.966, while accuracy increased to 0.903 ± 0.017. This result supports the value of explicitly representing the signed spectral changes between D2 and D5. The subsequent addition of the R branch further increased the AUC to 0.970 ± 0.013, suggesting that relative developmental changes provided information complementary to ΔS. Finally, the complete DSSF-Net with stage-aware attention achieved the highest numerical performance, with an accuracy of 0.915 ± 0.015, an AUC of 0.974 ± 0.012, and a precision of 0.916 ± 0.016.

Relative to direct D2 + D5 concatenation, the complete DSSF-Net produced numerical increases of 0.031 in accuracy, 0.023 in AUC, and 0.031 in precision. Along this sequential ablation pathway, the engineered developmental-change features ΔS and R contributed a cumulative AUC increase of 0.012, whereas the dual-stream encoder and stage-aware attention contributed a cumulative increase of 0.011. These numerical increments are consistent with contributions from the longitudinal developmental-change features and stage-specific fusion architecture. However, because each successive configuration also increased the number of trainable parameters, increased model capacity cannot be completely excluded as an explanation for part of the observed performance improvement.

### 3.6. Wavelength-Level Interpretation and Practical Implications

CARS and SPA were independently applied to the training data in each of the five outer folds. As shown in Table 7, CARS retained an average of 24.0 ± 1.6 wavelength positions, whereas SPA retained an average of 12.0 ± 0.7 positions, corresponding to approximately 3.5% and 1.8% of the original 681 sampling positions, respectively. SPA therefore produced a more compact candidate set, whereas CARS retained a larger number of wavelengths covering several local spectral regions that may provide complementary information.

Table 8 presents the results obtained using the unified outer five-fold evaluation procedure. The full-band DSSF-Net achieved an AUC of 0.974 ± 0.012, whereas the CARS- and SPA-based models achieved AUC values of 0.968 ± 0.014 and 0.961 ± 0.015, representing decreases of 0.006 and 0.013, respectively, relative to the full-band model. Both wavelength-selection schemes yielded numerically higher performance than their random-control models retaining the same numbers of wavelength positions. These findings indicate that the positions retained by CARS and SPA contained more predictive information than randomly selected positions of the same dimensionality.

In terms of dimensionality reduction, CARS achieved a numerical AUC close to that of the full-band model using only approximately 3.5% of the original wavelength positions. SPA further reduced the retained positions to approximately 1.8%, although this greater reduction was accompanied by a larger performance decrease. These results support the feasibility of wavelength dimensionality reduction but do not demonstrate that a very small number of wavelengths can fully replace the full-band system. Because the wavelength sets selected across the outer folds were not completely consistent and the reduced-band schemes have not yet been independently validated across incubation batches or measurement devices, the full-band DSSF-Net was retained as the final model in this study.

To avoid assessing wavelength stability based solely on a single selection performed using the complete dataset, the selection frequency of each sampling position across the five outer training sets was calculated. Table 9 presents only representative high-frequency positions. A frequency of 5/5 indicates that the corresponding position was selected by the respective method in all five outer training sets. Given that the 681 sampling positions were spaced at approximately 1 nm intervals, whereas the actual spectral resolution of the instrument was approximately 2.8 nm, adjacent positions should not be interpreted as independent and precisely resolved molecular absorption peaks. Instead, the selected positions should be summarized and interpreted at the spectral-region level.

**Figure 17:**
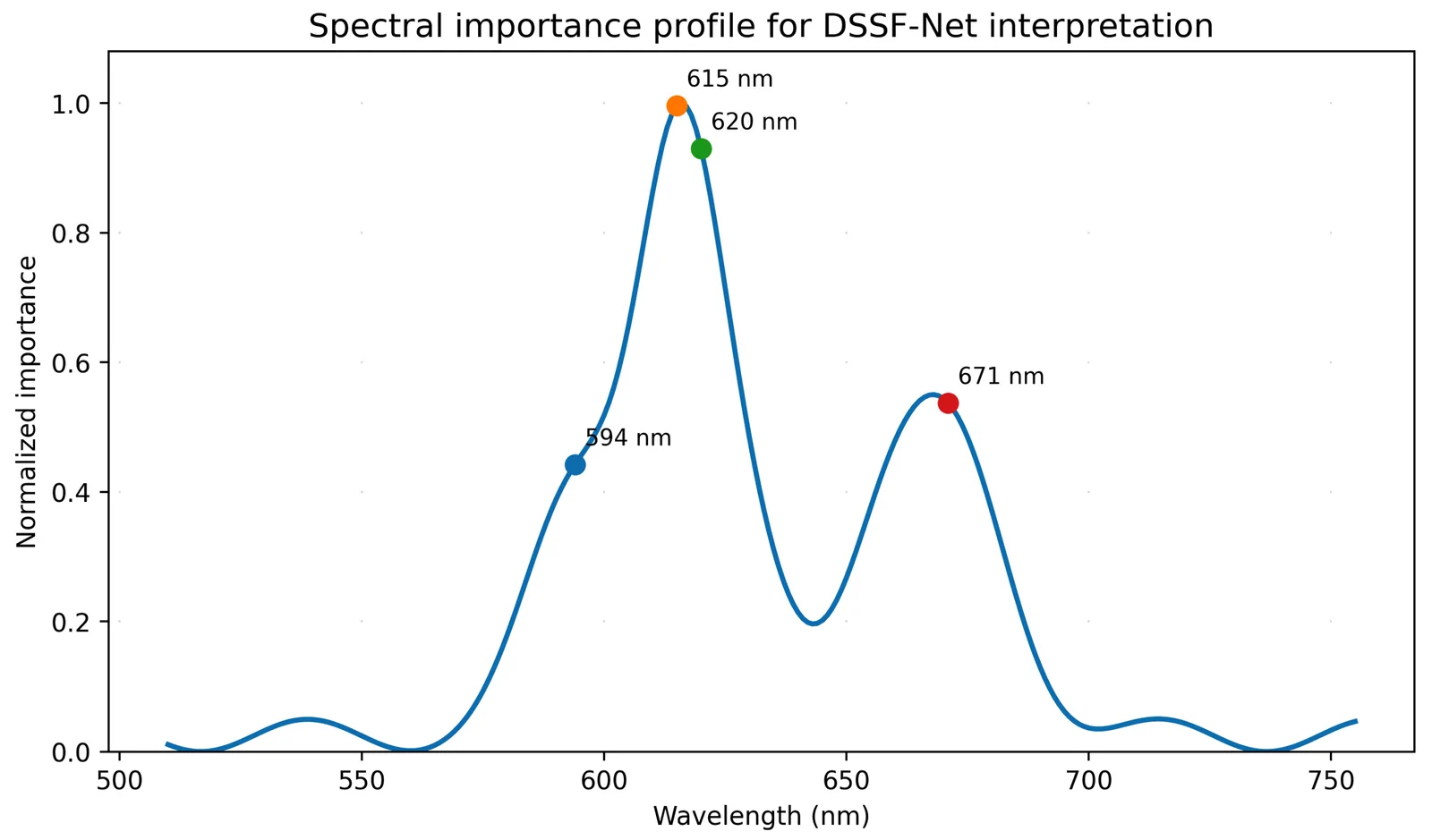
Composite wavelength-importance profile highlighting key regions within 510–755 nm.

As shown in Figure 17, relatively high model-derived importance was concentrated within the broader regions of 594–620 nm and 660–675 nm. Several local importance maxima were observed within these regions. Considering the approximately 2.8 nm spectral resolution, these maxima are interpreted as approximate positions within broader spectral regions rather than as independent features at individual 1 nm sampling points. Higher normalized importance values indicate greater contribution to discrimination. The model-derived importance regions partially overlapped with regions showing relatively large male–female spectral differences in Figure 8. This overlap provides internal consistency between the descriptive spectral analysis and the post-hoc model interpretation. As both analyses were based on the same dataset, this agreement is considered supportive internal evidence rather than independent biological validation, and its generalizability should be further examined using additional datasets and direct biological measurements.

From a general spectroscopic perspective, signals within the visible region can be influenced by wavelength-dependent hemoglobin absorption and changes in tissue absorption and scattering. Previous spectroscopic studies have reported distinct absorption characteristics of different hemoglobin states across the visible and near-infrared ranges, while tissue optical responses are jointly determined by absorbers and scattering structures. However, these references provide only a general spectroscopic basis and do not establish a direct correspondence between the model-derived regions and specific biological processes in chicken embryos. Because this study was based primarily on spectral data and post-hoc model interpretation, vascular density, hemoglobin concentration, pigmentation, hormone levels, histological development, and metabolic activity were not directly measured. Therefore, the identified regions should be interpreted as model-derived predictive wavelength regions rather than confirmed biological markers. Their possible biological relevance is presented only as a hypothesis for future investigation. Nevertheless, the analysis narrows the candidate spectral range and provides a useful basis for subsequent biological validation and the development of reduced-band multispectral detection systems.

The region-level comparison results are presented in Table 10. The 594–620 nm interval was identified as the primary candidate spectral range. Within this range, 611–620 nm exhibited the highest overall contribution, 601–610 nm showed a continuously increasing contribution, and 594–600 nm provided a secondary contribution. The 660–675 nm interval constituted another consistently important region, with a local maximum near 671 nm. The major and secondary peaks shown in Figure 17 were consistent with these region-level findings. Considering the instrumental spectral resolution of approximately 2.8 nm, the positions at 615 and 620 nm can be regarded as belonging to the same continuous high-contribution region, while those at 668 and 671 nm can be grouped within the same secondary contribution region.

The high importance observed near 620 and 671 nm was consistent with the preceding spectral-difference analyses of D2 and D5, respectively. Although 594 nm was particularly prominent in the D7 single-day analysis, this wavelength also exhibited a secondary contribution in the DSSF-Net model based on D2, D5, ΔS, and R inputs. In addition, CARS and SPA selected several variables near 434, 575, and 780 nm, indicating that spectral regions outside the principal candidate ranges may provide complementary information for model discrimination.

Overall, CARS and SPA produced only limited decreases in AUC despite substantially reducing the number of retained wavelength positions, and both yielded numerically higher performance than random controls of the same dimensionality. The SHAP and grouped-window ablation results from the full-band model also showed region-level agreement with the high-frequency positions selected by CARS and SPA. Collectively, these methods identified 594–620 nm and 660–675 nm as the principal candidate regions, with 611–620 nm demonstrating the strongest agreement across methods. These findings support the computational predictive value of the identified regions and provide a basis for subsequent wavelength simplification. However, their relationships with vascular density, hemoglobin concentration, tissue development, and metabolic activity require further validation using direct biological measurements. The feasibility of a dedicated multispectral system must also be evaluated with consideration of filter bandwidth, light-source stability, detection speed, batch-to-batch variation, and cross-device calibration.

In the present study, “non-destructive” refers strictly to an optical procedure that does not require eggshell penetration, egg opening, or biological-sample removal, rather than to a demonstrated absence of biological effects. Although the measured groups showed an overall descriptive hatchability rate of 92.8% among fertile eggs, no contemporaneous non-measured or minimally handled control group was included. Therefore, the potential effects of repeated handling, temporary removal from incubation, halogen illumination, and the approximately 2.3-h within-session acquisition window on embryonic development, mortality, or hatchability cannot be completely excluded. The fixed measurement order maintained approximately 12-h intervals for individual eggs but did not eliminate developmental-time differences between eggs measured at the beginning and end of each session. Future studies should include matched control groups and systematically monitor egg-surface temperature, cumulative illumination exposure, incubator recovery time, embryonic mortality, and hatchability.

## 4. Conclusions

This study systematically evaluated longitudinal Vis–NIR spectral information during incubation days 1–7 and identified D2 and D5 as two complementary early measurement windows for in ovo sex identification. Compared with single-day analysis or the direct inclusion of additional incubation days, D2 + D5 more effectively captured stage-specific information and developmental changes from the same egg. After the input scheme had been fixed, DSSF-Net achieved an AUC of 0.974 ± 0.012 under unified egg-level stratified five-fold cross-validation and an accuracy of 0.915 and an empirical AUC of 0.966 (95% CI: 0.940–0.987) on the fixed internal hold-out test set. These results are consistent with predictive contributions from the combination of stage-specific spectra, longitudinal developmental-change features, and staged fusion, although the possible contribution of the higher model capacity of DSSF-Net cannot be completely excluded.

Complete four-round leave-one-batch-out validation yielded a mean AUC of 0.926 ± 0.012, showing that the discriminative value of D2 + D5 was maintained across independent incubation batches within the investigated population. Post hoc wavelength-level interpretation further identified 594–620 nm and 660–675 nm as candidate spectral regions with relatively high predictive value. The agreement among spectral differences, wavelength-selection methods, SHAP analysis, and grouped-window ablation provides complementary computational evidence that the predictive information is concentrated within specific spectral regions rather than being uniformly distributed across the full spectral range. These findings provide useful wavelength guidance for subsequent biological investigation and reduced-band multispectral system development.

The present study established and systematically validated a longitudinal spectral modeling framework using fertilized pink-shelled eggs of Bashang Long-tailed chickens. Its broader applicability will be further evaluated across breeds, parental lines, shell colors, acquisition instruments, and incubation environments. Future research will also incorporate complete hatchery populations, direct biological measurements, automated egg tracking, online acquisition, throughput evaluation, and dedicated multispectral hardware validation. Overall, the identification of the D2 + D5 combination, the demonstrated value of longitudinal developmental-change features, and the consistent results across fixed independent testing and four incubation batches provide a strong experimental and methodological foundation for advancing early non-destructive in ovo sex identification toward automated hatchery applications.

## Figures and Tables

**Table 1:** Model Parameter Settings.

Item	Setting
Framework	Python 3.7.6 + PyTorch 1.13.1
Optimizer	Adam
Initial learning rate	1.0 × 10^−3^
Weight decay	1.0 × 10^−4^
Batch size	32
Maximum epochs	200
Early-stopping patience	20
Dropout rate	0.3
Random seed	42
Learning-rate scheduler	factor = 0.5, patience = 8

**Table 2:** Model-specific architecture.

Model	Actual Model-Specific Architecture
1D-CNN	Input: 4 × 681; three Conv1D blocks with 32, 64, and 128 channels; kernel sizes: 7, 5, and 3; each block followed by batch normalization, ReLU, and max pooling (kernel = 2, stride = 2); adaptive average pooling; FC: 128→2
BiLSTM	Input sequence: 681 × 4; two bidirectional LSTM layers; hidden size = 64 per direction; dropout = 0.3; concatenated forward and backward features; FC: 128→2
Transformer Encoder	Input projection: 4→128; two encoder layers; four attention heads; d_model = 128; feed-forward dimension = 256; dropout = 0.3; global average pooling; FC: 128→2
DSSF-Net	Two independent stage-specific encoders for D2 and D5, each containing three residual Conv1D blocks with 32, 64, and 128 output channels and kernel sizes of 7, 5, and 3, respectively. Each block included batch normalization, ReLU activation, max pooling (kernel = 2, stride = 2), and a projection shortcut implemented using 1 × 1 convolution and corresponding downsampling. Adaptive global average pooling generated a 128-dimensional feature vector from each branch.

**Table 3:** Exploratory validation performance of representative single-day and multi-day spectral combinations under the fixed data partition.

Days	Best Combination	Accuracy	AUC	Median AUC	F1-Score
2	D2 + D5	0.885	0.951	0.771	0.886
3	D2 + D5 + D6	0.880	0.950	0.807	0.882
4	D2 + D3 + D5 + D6	0.875	0.938	0.821	0.876
5	D1–D5	0.870	0.920	0.835	0.871
6	D1–D6	0.862	0.912	0.898	0.863
7	D1–D7	0.855	0.905	0.905	0.856

**Table 4:** Classification performance of different models based on five-fold cross-validation.

Model	Accuracy	AUC	Precision	Recall	F1-Score
PLS-DA	0.755 ± 0.031	0.827 ± 0.025	0.756 ± 0.029	0.753 ± 0.034	0.754 ± 0.030
SVM	0.835 ± 0.024	0.904 ± 0.018	0.836 ± 0.026	0.834 ± 0.023	0.835 ± 0.024
RF	0.860 ± 0.022	0.912 ± 0.019	0.861 ± 0.020	0.859 ± 0.024	0.860 ± 0.021
XGBoost	0.885 ± 0.020	0.916 ± 0.017	0.886 ± 0.022	0.884 ± 0.018	0.885 ± 0.019
1D-CNN	0.895 ± 0.018	0.931 ± 0.016	0.896 ± 0.017	0.894 ± 0.020	0.895 ± 0.017
BiLSTM	0.905 ± 0.017	0.941 ± 0.015	0.906 ± 0.018	0.904 ± 0.015	0.905 ± 0.016
Transformer Encoder	0.890 ± 0.019	0.956 ± 0.014	0.891 ± 0.021	0.889 ± 0.017	0.890 ± 0.018
DSSF-Net	0.915 ± 0.015	0.974 ± 0.012	0.916 ± 0.016	0.914 ± 0.014	0.915 ± 0.014

**Table 5:** Confirmatory comparison of four predefined acquisition and modeling configurations on the fixed internal hold-out test set.

Configuration	Accuracy	AUC (95% CI)	Sensitivity	Specificity	Precision	F1-Score
D5-only baseline	0.790	0.865 (0.815–0.908)	0.780	0.800	0.796	0.788
Direct D2 + D5 baseline	0.875	0.943 (0.909–0.969)	0.880	0.870	0.871	0.876
D2 + D5 + ΔS + R with non-attentive fusion	0.905	0.960 (0.932–0.980)	0.900	0.910	0.909	0.905
Full DSSF-Net with stage-aware attention	0.915	0.966 (0.940–0.987)	0.910	0.920	0.919	0.915

**Table 6:** Sequential ablation results of DSSF-Net components under unified five-fold cross-validation.

Configuration	Accuracy	AUC	Precision
Direct D2 + D5 concatenation	0.884 ± 0.021	0.951 ± 0.017	0.885 ± 0.020
+Dual-stream encoder	0.891 ± 0.019	0.958 ± 0.015	0.893 ± 0.018
+Difference spectrum ΔS	0.903 ± 0.017	0.966 ± 0.014	0.905 ± 0.016
+Relative change rate R	0.909 ± 0.016	0.970 ± 0.013	0.911 ± 0.015
DSSF-Net	0.915 ± 0.015	0.974 ± 0.012	0.916 ± 0.016

**Table 7:** Numbers of Wavelength Positions Retained by CARS and SPA across the Five Outer Training Folds.

Method	Fold 1	Fold 2	Fold 3	Fold 4	Fold 5	Mean ± SD
CARS	24	23	26	22	25	24.0 ± 1.6
SPA	12	11	13	12	12	12.0 ± 0.7

**Table 8:** Outer Five-Fold Classification Performance Using Different Wavelength Input Schemes.

Input Scheme	Number of Retained Wavelength Positions	Accuracy	AUC	F1-Score
Full-band	681	0.915 ± 0.015	0.974 ± 0.012	0.915 ± 0.014
CARS-selected	24.0 ± 1.6	0.909 ± 0.017	0.968 ± 0.014	0.909 ± 0.016
SPA-selected	12.0 ± 0.7	0.900 ± 0.019	0.961 ± 0.015	0.900 ± 0.018
Random-k (Matched to CARS)	Matched within Each Fold	0.874 ± 0.024	0.928 ± 0.022	0.873 ± 0.024
Random-k (Matched to SPA)	Matched within Each Fold	0.852 ± 0.027	0.906 ± 0.025	0.851 ± 0.027

**Table 9:** Selection Frequencies of Representative Sampling Positions across the Five Outer Training Sets.

Sampling Position (nm)	CARS Selection Frequency	SPA Selection Frequency	Candidate Spectral Region
594	5/5	4/5	594–620 nm
602	4/5	4/5	594–620 nm
610	5/5	4/5	594–620 nm
615	5/5	5/5	594–620 nm
620	5/5	5/5	594–620 nm
660	4/5	4/5	660–675 nm
668	5/5	5/5	660–675 nm
671	5/5	5/5	660–675 nm
675	4/5	4/5	660–675 nm
434	3/5	3/5	Other Spectral Regions
575	3/5	3/5	Other Spectral Regions
780	2/5	3/5	Other Spectral Regions

**Table 10:** Region-Level Agreement among Wavelength-Selection and Interpretation Methods.

Candidate Spectral Region	Normalized SHAP Importance	Normalized Ablation Importance	Representative CARS Evidence	Representative SPA Evidence
594–600 nm	0.46	0.42	594 nm: 5/5	594 nm: 4/5
601–610 nm	0.78	0.74	602: 4/5; 610: 5/5	602, 610: 4/5
611–620 nm	1.00	0.96	615, 620: 5/5	615, 620: 5/5
660–667 nm	0.49	0.46	660 nm: 4/5	660 nm: 4/5
668–675 nm	0.56	0.52	668, 671: 5/5; 675: 4/5	668, 671: 5/5; 675: 4/5

## Data Availability

The datasets generated and analyzed during the current study are not publicly available because they form part of an ongoing research project and are subject to institutional confidentiality and data-management requirements. Requests to access the datasets should be directed to the corresponding author and will be considered for legitimate academic purposes, subject to institutional approval and applicable confidentiality restrictions.
